# Copper Selenides via Anion Exchange versus Direct Growth – The Role of Diorganyl Diselenides

**DOI:** 10.1021/acs.inorgchem.5c04328

**Published:** 2025-11-14

**Authors:** Jiwoo Choi, Benjamin A. Schmidt, Mykhailo Boleychuk, Kiran Bedi, Emily Sandoval-Arteaga, Kezia N. Almonte, Quentin M. Boussard, J. Kenneth Krebs, Malgorzata Kowalik, Adri van Duin, Katherine E. Plass

**Affiliations:** a Department of Chemistry, Franklin & Marshall College, Lancaster, Pennsylvania 17604, United States; b Department of Physics, Franklin & Marshall College, Lancaster, Pennsylvania 17604, United States; c Department of Mechanical Engineering, 8082 Pennsylvania State University, University Park, Pennsylvania 16802, United States

## Abstract

Anion exchange is a powerful tool for postsynthetic transformation of nanoparticles that can be coupled with other transformations to create nanostructures unachievable by direct growth. Previous work implicated long-chain dialkyl diselenides as drivers of Se^2–^ anion exchange on Cu_2–*x*_S, but it lacked mechanistic details. Here, we examined the reactivity trends of diphenyl, didodecyl, and dibenzyl diselenides as drivers of anion exchange to create metastable copper sulfur selenide alloys. We contrasted these reactivity trends with those of direct synthesis of copper selenide nanoparticles, demonstrating mechanistic orthogonalities between these pathways. Dialkyl diselenides were the only species that induced anion exchange. We rationalized the different reaction outcomes using thermal decomposition measurements together with MD simulations using a ReaxFF force field (trained against DFT data). Dialkyl diselenides were shown to release H_2_Se, which appears to be critical for achieving Se^2–^ anion exchange. This demonstrates the utility of computationally inexpensive atomistic-scale simulations methods like ReaxFF in the rational design of nanoparticle syntheses. Using this insight, the dialkyl diselenides were identified as a new class of selenium exchange reagents based on initial MD simulations of H_2_Se release. This work provides mechanistic understanding of nanoparticle anion exchange and insights into decomposition processes crucial to use of diorganyl diselenides in nanoparticle synthesis.

## Graphical abstract

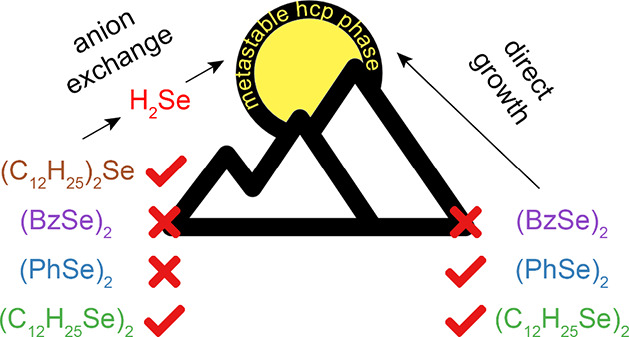

## Introduction

Molecular-level insights into nanoparticle growth and postsynthetic transformation processes are essential to achieve rational design over phase, composition, size, and shape.^1–4^ Though it remains challenging to uncover mechanisms behind direct nanoparticle growth, numerous early investigations showed the power of the approach,^5–12^ and more recent work has built on this to demonstrate extensive phase control over particular systems.^13–16^ Postsynthetic transformation (PST) of nanoparticles, like cation and anion exchange, etching, and seeded growth, can create complicated nanomaterials through tailoring shapes, crystalline phases, and composition,^17–20^ complementing direct nanoparticle synthesis. One useful approach for gaining mechanistic insights is to compare starting reagents with similar structures to reveal reactivity trends, a widespread approach to understanding nanoparticle growth^13,21–25^ that has been less frequently applied to understanding PST.^26^ Anion exchange is a specific PST that has proven useful in selenizing or tellurizing a variety of binary and ternary metal sulfides.^27–33^ Here, we examine reactivity trends to understand selenium anion exchange on copper sulfides.^28,31–33^ We also establish the use of simulations with the ReaxFF potential^34–36^ as a means of rationalizing and predicting mechanistic behavior with low computational power. Copper chalcogenides and their alloys are of interest for applications that employ their ion storage and transport (like cation and anion exchange), photothermal, thermoelectric, photocatalytic, and plasmonic properties.^37^ They are also synthons for a variety of different metal chalcogenides with a controlled phase, regioselectivity, shape, and size. In these applications, phase selectivity is crucial; synthesis of phase-pure metal chalcogenides is, however, complicated by many factors that need to be appropriately tuned, as reviewed by Endres et al.^38^ The copper selenide system, for example, consists of numerous compositions, crystal systems, and polymorphs that can be selected by variation of reaction time, temperature, selenium precursor, and solvent conditions.^16,39–41^ The connection between phase selection and reaction conditions, however, is often opaque and can be complicated by solvent–ligand interactions, for example.^42^ Mechanistic understanding of such phase-selective creation has broad consequences for creating new nanomaterial design tools.

The diorganyl dichalcogenide system (R_2_E_2_, where E = S, Se, or Te) provides an intriguing opportunity to develop our mechanistic understanding of postsynthetic transformations while making comparisons with a direct growth of metal chalcogenide nanoparticles. Diorganyl dichalcogenides are a versatile class of precursors with well-studied reactivity trends for phase-selective nanoparticle synthesis. They allow phase-selective direct synthesis of various metal chalcogenide nanocrystal phases^13,16,23,39,43–50^ and form nanocrystal surface ligands,^51,52^ as first uncovered by Guo et al.^43^ and reviewed by Brutchey.^53^ Their E-E bonds may be readily cleaved to generate a chalcogenide source whose reactivity can be tuned by the attached organyl group.^53^ Using this tunability, the rate of chalcogenide release can be altered to selectively synthesize metal chalcogenides with either thermodynamic or metastable phases. Rapid C–Se bond breaking releases Se to promote the growth of thermodynamically favorable phases, while stronger C–Se bonds slow the release of Se, and therefore its reaction with Cu precursors, to enable interactions that favor the formation of metastable phases. Intriguingly, dialkyl diselenide has been found to play a key role in the recently discovered selenium anion exchange of roxbyite Cu_2–*x*_S nanorods to create nanorods of Cu_2–*x*_(S_,_Se) solid solutions.^32^ This insight, however, left us puzzled as to the action of dialkyl diselenides in promoting anion exchange. Did the dialkyl diselenide directly bind to the Cu_2–*x*_S nanorod surface and then release Se^2–^ that could diffuse into the particle? Or was further decomposing into a solution species that had direct action on the particles? It also raised the question of whether long-chain dialkyl diselenides could be replaced by commercially available diselenides, such as diphenyl diselenide or dibenzyl diselenide. The existing knowledge regarding the use of diorganyl diselenides to grow the metal selenide nanoparticle phase selectively could be a useful basis with which to understand their ability to induce Se^2–^ anion exchange and create useful copper chalcogenide materials. This provides an opportunity to better understand the driving forces behind the selenization of metal sulfides while also comparing direct synthesis and anion exchange in the same copper sulfide/selenide system.

Numerous diselenides promote the synthesis of various metal selenide nanoparticle phases;^43,53,54^ we chose to compare didodecyl diselenide, diphenyl diselenide, and dibenzyl diselenide^41,44,47,53^ (Scheme 1). This choice allowed a comparison of three species with distinctly different relative bond strengths and demonstrated the ability to generate different phases. Furthermore, these three compounds allowed direct comparison with previous work, were straightforward to obtain, and were safer to work with than diselenides that are stench compounds. Diphenyl diselenide ((PhSe)_2_) and dialkyl diselenides have promoted metastable hexagonal phase formation in CuInS_2_,^46^ Cu_2–*x*_Se,^16,41,44^ Cu_2–*x*_(S,Se) alloys,^50^ and copper tin selenides.^47^ Dibenzyl diselenide ((BzSe)_2_), however, promotes the thermodynamically preferred cubic phase in CuInS_2_.^46^ Phase mapping of copper selenide synthesis revealed how particular combinations of dichalcogenide precursors, reaction time, temperature, and oleylamine/octadecene ratios altered the phase and phase purity of the copper selenide produced. This mapping identified a few conditions under which (PhSe)_2_ can produce cubic phases but many conditions in which it creates an hexagonal phase; (BzSe)_2_ only produced cubic phases.^16^ When selecting the dialkyl diselenides to use, we eschewed the short-chain dialkyl diselenides like diallyl diselenide and di-*tert*-butyl diselenide that have been used for phase-selective synthesis in favor of long-chains.^43,53,54^ We chose to use didodecyl diselenide based on the demonstration that didecyl diselenide causes Se^2–^ exchange on roxbyite Cu_2–*x*_S to generate a metastable wurtzite phase alloy^32^ and didodecyl diselenide allows direct synthesis of the same phase of Cu_2–*x*_Se.^44^ The long-chain dialkyl diselenides also have a lower volatility that reduces their stench and increases the ease with which they can be purified by recrystallization.

**Scheme 1:**
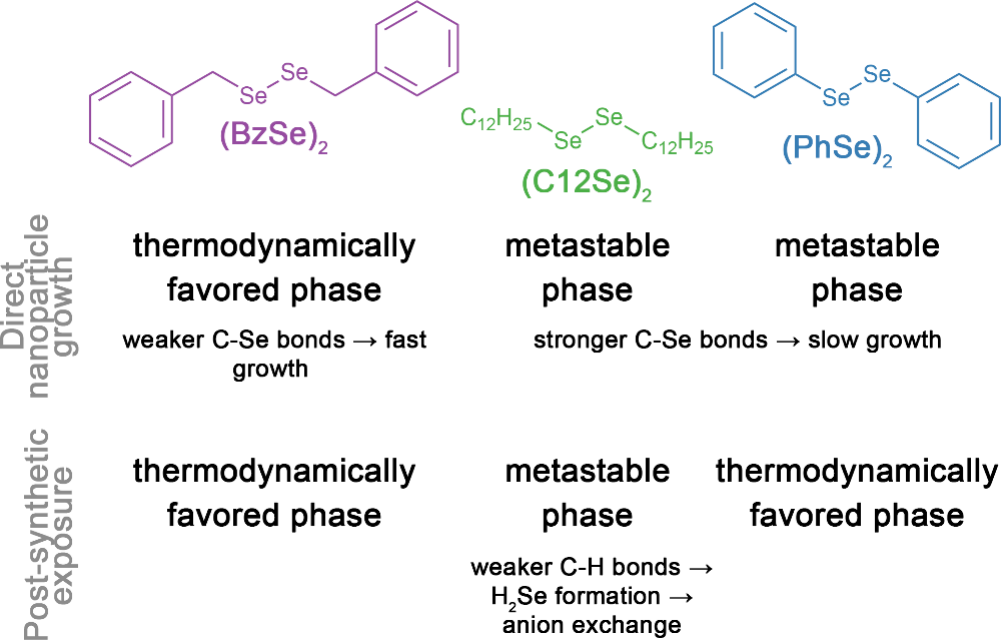
Postsynthetic Transformations of Cu_2–*x*_S Nanorods Are Investigated Using the Three Diorganyl Diselenide Molecules Shown^a^

a The phase generated when the diselenide is used for direct growth of metal selenide nanoparticles^41,44,47,50,53^ versus selenium anion exchange to demonstrate the different reactivity trends observed.

Inspired by the reactivity trends of diorganyl diselenides in directing phase-selective metal selenide nanoparticle growth, we seek to develop and rationalize an analogous reactivity trend for the selenization of copper sulfide by anion exchange. We thus attempted anion exchange on roxbyite Cu_2–*x*_S nanorods using the same conditions developed for didecyl diselenide^32^ and evaluated the resultant particles using PXRD, TEM, and SEM-EDS. Molecular dynamics (MD) simulations were performed following a comparison of ReaxFF and DFT C–Se and Se–Se bond dissociation energy (BDE) calculations on various diorganyl diselenide molecules to vet the newly developed force field. We rationalized the observed reactivity trend by using ReaxFF to model the thermal decomposition of diorganyl diselenides and ensured the validity of the simulation by comparison with the experiment. Using our molecular dynamics inspired hypothesis, we were able to propose and successfully test a new class of selenizing agents, the dialkyl monoselenides.

## Experimental Section

### Chemicals

The reagents used for the postsynthetic transformation and alkyl selenide syntheses included selenium (powder, −100 mesh, ≥99.5% trace metals basis), sodium borohydride (ReagentPlus, 99%), diphenyl diselenide (98%), dibenzyl diselenide (95%), oleylamine (technical grade, 70%), 1-octadecene (technical grade, 90%), sodium borohydride (%), 1-bromododecane (98%), 1-bromohexadecane (97%), hexadecyltrimethylammonium bromide (≥98%), tetrahydrofuran (THF) (≥99%, dried in solvent column), and magnesium sulfate (97%). Analytical grade solvents used included ethanol (200 proof, Greenfield Global), isopropyl alcohol, toluene, heptane, and acetone. Deuterated chloroform (D, 99.96%) was purchased from Cambridge Isotope Laboratories, Inc. Unless specified, all reagents were purchased from Sigma-Aldrich and used as received.

### General Safety Concerns

The synthetic methods are performed under air-free conditions at elevated temperatures by using high-boiling-point solvents. As such, care should be taken to ensure proper monitoring and handling. For example, burns have been reported from exposure to high-temperature oleylamine.^55^ The synthesis of didodecyl diselenide, the selenization process, and thermal decomposition all have the potential to release volatile, toxic selenium compounds. Ensure proper containment and venting. This synthesis also requires sodium borohydride, which reacts vigorously with ethanol and water to release flammable H_2_ gas. Care must be taken to avoid overpressurization and to avoid fire. The safety data sheets for all chemicals used in the reactions should be reviewed, and proper personal protective equipment should be used.

### Standard Reaction Vessel Setup

Each of the following procedures utilized either a standard Schlenk line setup or an Ar gas manifold. Each dried, three-necked, round-bottom flask was equipped with a magnetic stir bar, a reflux condenser with a glass adaptor connected either to the Schlenk line or to a bubbler containing mineral oil (in the middle neck of the three-necked flask), a thermocouple inserted through a silicone septum on one side neck, and a second septum with a needle connected to the Ar gas on the other side neck. The temperature was controlled by heating mantles on magnetic stir plates.

### Synthesis of Didodecyl Diselenide (C_12_Se)_2_

First, disodium diselenide was synthesized in an alkaline aqueous solution as described by Lim et al.^56^ Note that this route claims to avoid H_2_Se production during the synthesis. Selenium (3.95 g, 50 mmol) was added to an alkaline aqueous solution (25 mL) that contained NaOH (2.0 g, 50 mmol) and hexadecyltrimethylammonium bromide in a catalytic amount (a small scoop). Then, NaBH_4_ (0.25 g, 6.6 mmol) with NaOH (0.2 g, 5 mmol) in 5 mL of water was cooled in an ice bath and then added dropwise at room temperature under an argon atmosphere with stirring. The mixture was stirred for 60 min at room temperature, then raised to 90 °C, and stirred at this temperature for 30 min to complete the reaction. A brownish-red aqueous solution of Na_2_Se_2_ was obtained.

1-Bromododecane (12 mL, 50 mmol) and tetrahydrofuran (60 mL) were added to the Na_2_Se_2_ solution dropwise for 30 min under vigorous stirring. The reaction was then allowed to react at 50 °C for at least 18 h until the solution turned a yellow-orange color and then cooled down to room temperature. Following cooling, the phases were separated. The organic layer was washed once with deionized H_2_O, and then, the combined organic layers were washed with brine and dried over magnesium sulfate. The solvent was removed under reduced pressure, and the crude product was recrystallized from heptane to yield yellow, needle-shaped crystals. Multiple recrystallizations were needed to achieve purity according to NMR. If the solid started to appear red, it was repurified before use. Identity was verified using 1H and 77Se NMR.^44^ 1H NMR (400 MHz, CDCl3) δ 2.92 (t, 4H), 1.73 (q, 4H), 1.27 (m, 36H), 0.88 (t, 3H) ppm. 77Se (76 MHz, CDCl3), δ 307.6 ppm. Note that these matched reported values for didodecyl diselenide.^44^

### Synthesis of Dihexadecyl selenide (C_16_)_2_Se

The synthesis of dihexadecyl monoselenide closely followed the procedure described above, with minor revisions. The alkaline aqueous solution was prepared with a double amount of NaBH_4_ (0.50 g, 13.2 mmol) and stirred for approximately 18 h with the same 30 min heating period to give a dark brown-red mixture. 1-Bromohexadecyl (30.5 mL, 100 mmol, double the amount from the synthesis of the diselenide) in THF (100 mL) was introduced portion-wise into the Na_2_Se solution over 30 min, changing the solution color to gray-green. The reagents were left to react for 3 h, following which the resulting solution was worked-up (same as [C12–Se]_2_) and recrystallized repeatedly (at least 4 times) to yield white crystals. ^1^H NMR (400 MHz, CDCl_3_) δ 2.54 (t, 4H), 1.65 (q, 4H), 1.26 (m, 36H), 0.88 (t, 3H) ppm. ^77^Se (76 MHz, CDCl_3_), δ 160.25 ppm. Note that these matched reported values for didecyl selenide.^57^

### Selenium Transformation with Diorganyl Selenides

Selenium transformation was carried out using diorganyl diselenides (didodecyl diselenide, diphenyl diselenide, or dibenzyl diselenide) or dihexadecyl selenide by adapting the previously reported procedure^32^ as follows. Cu_2–*x*_S nanorods were synthesized as previously reported.^32,58^ Didodecyl diselenide (0.0745 g, 0.20 mmol) was dissolved in 10 mL of octadecene and added to the standard reaction setup and flushed with Ar. All diselenides used 0.20 mmol (0.40 mmol of Se), while dihexadecyl selenide employed 0.30 mmol overall (0.30 mmol of Se). The reaction was heated to various temperatures between 100 and 260 °C, followed by swift injection of suspended nanoparticles (20 ± 4 mg) in 4.0 mL of oleylamine. The reaction was stirred for 120 min at constant temperature (±4 °C) and then cooled to room temperature using an ice water bath. The solution was transferred to a 50 mL centrifuge tube, and ethanol (20 mL) was added. The solution was centrifuged (5 min at 6000 rpm). Washing and centrifugation were repeated using a 4:1 ratio of ethanol to heptane (5 min at 6000 rpm).

### Thermal Decomposition of Diorganyl Diselenides

The procedure was modeled on that reported by Koziel et al.^39^ Each diorganyl diselenide (0.05 mmol) was placed in an NMR tube capped by a new rubber septum and purged with Ar gas. Samples were heated to 260 °C in a sand bath for 2 h with an Ar balloon to maintain back pressure. After cooling, 0.60 mL of CDCl_3_ was added to each tube via syringe to maintain a constant concentration.

### Molecular Dynamics Simulations

Molecular dynamics (MD) simulations were conducted using the newly developed ReaxFF^34–36^ force field on dibenzyl diselenide, didodecyl diselenide, didecyl diselenide, dodecyl selenide, and diphenyl diselenide using the Amsterdam Modeling Suite (AMS 2023 or AMS 2024)^59,60^ on the Franklin & Marshall Computational Cluster. All constant pressure simulations (NPT, where N is a constant number of molecules, P is a constant pressure, and T is a constant temperature) were initially run at 100 K and 100 Pa with a damping constant of 100 fs to densify the models composed of 50 molecules of the given type of diorganyl diselenide with the specified final density (didecyl selenide 1.29 g/mL, didecyl diselenide 1.35 g/mL, dibenzyl diselenide 1.87 g/mL, didodecyl diselenide 1.16 g/mL, or diphenyl diselenide 1.86 g/mL). All elevated temperature simulations (1000 K, 1500 K, and 2000 K) were performed at constant volume (NVT, where N is a constant number of molecules, V is a volume, and T is a temperature) with a Berendsen thermostat and 100 fs damping constant. These elevated temperature simulations were performed for 2,000,000 steps with a 0.25 fs time step to give an overall simulation time of 500 ns. The Movie feature in AMS software allowed for a graphical visualization and the implemented ChemTraYzer 2 reaction detection algorithm for the statistical analysis of the identified reactions. Also, the graphs showing a time evolution of the number of molecules of the given type were obtained with the Movie feature. We chose to plot Se-containing species that made up at least 1% of the total molecules on average (Tables S4, S5).

## Results and Discussion

### Establishing Reactivity Trends and Comparison with Direct Growth

To assess which diorganyl diselenide molecules induced the Se^2–^ anion exchange of Cu_2–*x*_S nanoparticles, we injected roxbyite Cu_2–*x*_S nanorods into a solution of each of the three diorganyl diselenide molecules presented in Scheme 1, didodecyl diselenide (C_12_Se)_2_, dibenzyl diselenide (BzSe)_2_, and diphenyl diselenide (PhSe)_2_. Initially, we hypothesized that (PhSe)_2_ and (C_12_Se)_2_ would promote anion exchange for the same reason that they promote the direct synthesis of metastable phases—their relatively strong C–Se bonds produce slow-reacting organyl Se-species, which can have strong surface interactions (Scheme 1).^52^ As such, those species could bind to the Cu_2–*x*_S surface, release Se in a form that would diffuse into the Cu_2–*x*_S nanorods, and displace S^2–^ ions. Correspondingly, because (BzSe)_2_ rapidly releases reactive selenium species and promotes direct growth of the thermodynamically preferred phase, it may dissolve Cu_2–*x*_S and then react with released Cu^+^ to form Cu_2–*x*_Se. To test this hypothesis, these diorganyl diselenides were dissolved in 1-octadecene and particles were injected from a 1-oleylamine suspension at various temperatures (100, 150, 180, and 260 °C are presented here). These temperatures were chosen based on our previous observations that anion exchange occurs at 260 °C.^32^ Key markers of anion exchange were incorporation of Se (as measured by SEM-EDS), preservation of the nanorod size and morphology (as measured by TEM), and retention of the quasi-hexagonally close-packed anion sublattice to form a wurtzite phase, as opposed to the more thermodynamically favorable cubic berzelianite phase (as measured by PXRD).

#### Postsynthetic Transformation by Dibenzyl Diselenide (BzSe)_2_

Dibenzyl diselenide (BzSe)_2_ does not promote anion exchange, which is consistent with our direct-growth-inspired hypothesis and allows us to start to rationalize when PST would be outcompeted by dissolution processes. PST of roxbyite nanorods with dibenzyl diselenide (BzSe)_2_ resulted in the dissolution and reprecipitation of the klockmannite phase^16^ at lower temperatures (150 and 180 °C) and the thermodynamically preferred berzelianite phase at higher temperatures (260 °C), as supported by phase, morphology, and levels of Se-incorporation into the resultant particles (Figure 1, Table S1). After 2 h of exposure to (BzSe)_2_ at 100 °C, the roxbyite Cu_2–*x*_S particles show little change. The PXRD pattern (Figure 1a) shows a slight shift in the prominent reflections at 46 and 49° 2θ, likely due to a phase shift to a more Cu-rich phase as the observed Se/S ratio is near zero (Table S1, 0.07 ± 0.01). TEM shows that the nanorod shape remains, although the particles are less well-dispersed, indicating that dissolution may be occurring (Figure 1b). After exposure at 150 and 180 °C, a transformation to klockmannite nanoplates is observed. The PXRD (Figure 1a) shows key reflections at 28, 31, 47, and 51° 2θ, coupled with an absence of peaks that would indicate wurtzite Cu_2–*x*_(S,Se) or berzelianite Cu_2–*x*_Se. The particles have become heterogeneous platelets, often adopting a hexagon shape (Figure 1b). Notably, growth conditions have been created similar to those identified by Williamson et al. through machine-learning strategies,^16^ wherein an optimal synthesis of klockmannite is found to use the (BzSe)_2_ precursor (203.5–234.3 °C with <9.3 vol % of oleylamine in 1-octadecene for 22.5– 30.4 min), though lower temperature and longer times are used here. After exposure at high temperature (260 °C), the Cu_2–*x*_S nanorods fully dissolve and precipitate as cubic berzelianite. PXRD (Figure 1a) shows only reflections that coincide with the most intense peaks of the thermodynamically preferred berzelianite phase (37, 45, 52° 2θ). TEM images (Figure 1b) show only large, poorly formed particles, indicative of rapid growth. The observed dissolution and reprecipitation processes are consistent with observations from direct growth of Cu_2_SnSe_3_ ^47^ and CuInSe_2_,^46^ in which the weak C–Se bond of (BzSe)_2_ quickly breaks and creates reactive species like Se(0) and dibenzyl triselenide.^39^ Upon thermal decomposition of (BzSe)_2_, these reactive Se precursors dissolve the Cu_2–*x*_S particles, freeing Cu^+^ to reform new Cu_2–*x*_Se nanoparticles. Release of highly reactive Se species would both promote the growth of thermodynamically preferred phases and discourage PST.

**Figure 1:**
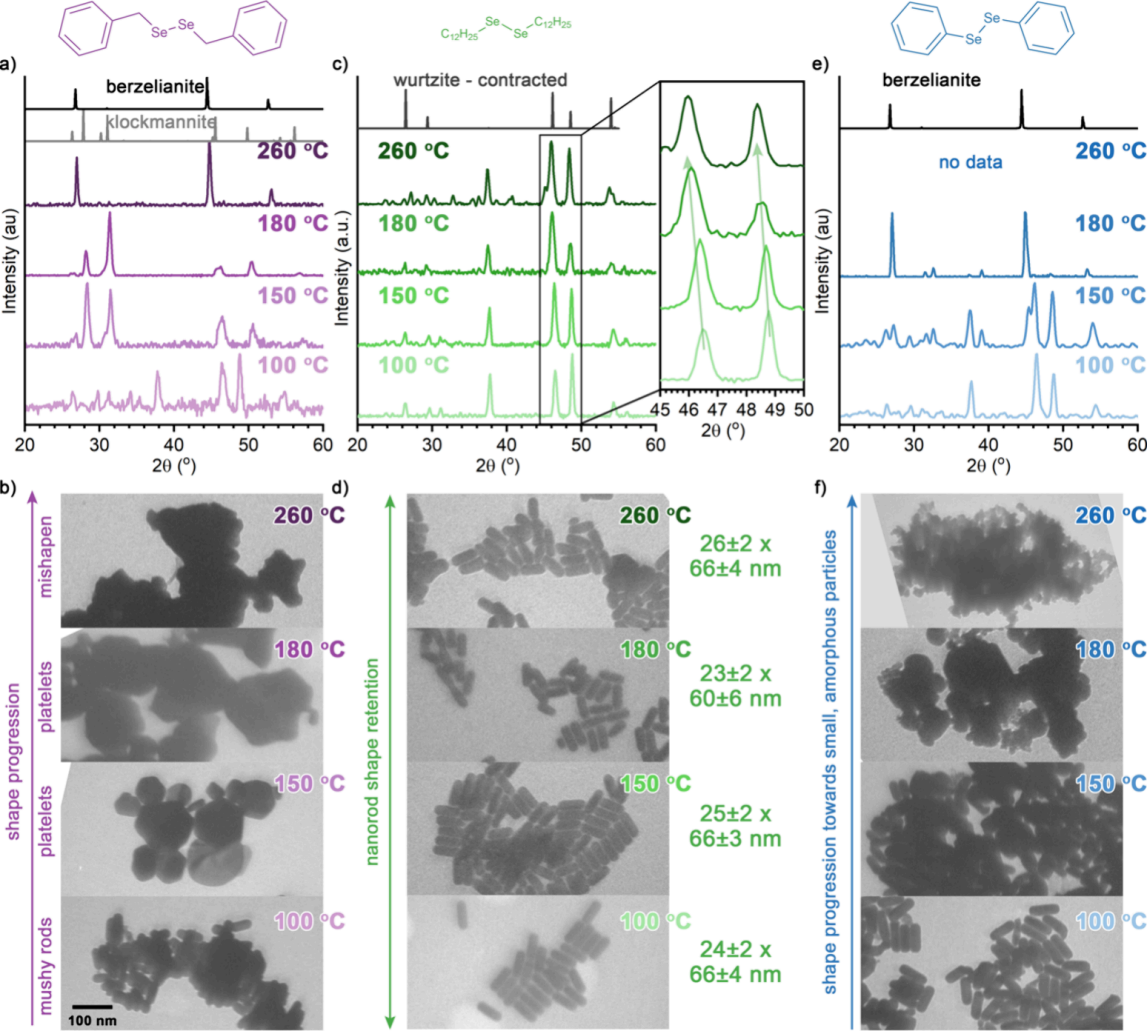
Results of postsynthetic transformation of Cu_2–*x*_S nanorods using (BzSe)_2_ (purple, left), (C_12_Se)_2_ (green, center), and (PhSe)_2_ (blue, right) at various temperatures. (a,c,e) PXRD shows the evolution of the crystalline phase with increasing temperature. Only (C_12_Se)_2_ retained the hexagonally close-packed (hcp) wurtzite phase,^44^ indicative of anion exchange (c), while (BzSe)_2_ (a) and (PhSe)_2_ (e) both dissolved Cu_2–*x*_S and precipitated ccp klockmannite (ICSD82331)^61^ or berzelianite phases (ICSD41140).^62^ (b,d,f) TEM shows the evolution of particle morphology. Reinforcing the observations from PXRD, only (C_12_Se)_2_ retained the nanorod length and width, indicative of anion exchange, as shown by the size comparison.

#### Postsynthetic Transformation by Didodecyl Diselenide (C_12_Se)_2_

Didodecyl diselenide, (C_12_Se)_2_, drives anion exchange at a variety of temperatures, extending earlier observations showing that long-chain diselenides were effective at 260 °C.^32^ Note that we have employed both didodecyl diselenide (here) and didecyl diselenide^32^ and both promote anion exchange at 260 °C. (C_12_Se)_2_ induces Se^2–^ anion exchange across the temperatures evaluated, where higher temperatures show an increased extent of Se incorporation and greater shifts in the position of the PXRD, indicating Cu_2–*x*_(S,Se) solid solution formation. Notably, anion exchange with such long-chain dialkyl diselenides broadens the temperature range where anion exchange with Se metal is inaccessible due to competing dissolution/reprecipitation pathways caused by selenides species.^32^ Injection of nanorods suspended in oleylamine into a (C_12_Se)_2_/1-octadecene mixture at different temperatures (100, 150, 180, and 260 °C) resulted in retention of the hexagonally close-packed anion sublattice (Figure 1c, Figures S1–S6) as well as the nanorod morphology (Figure 1d and Figure S7). PXRD (Figure 1c) shows peaks consistent with a solid-solution of the hcp Cu_2–*x*_Se wurtzite lattice with the quasi-hcp Cu_2–*x*_S roxbyite lattice (see the detailed justification and comparison of crystal structures in Figures S1–S6). The peaks between 45 and 50° 2θ shift to smaller values as the temperature of the exchange increases, indicating increasing amounts of selenium ions, which expand the initial Cu_2‑x_S lattice. Notably, the PXRD is quite phase-pure between 100 and 180 °C, but a berzelianite impurity is indicated by a peak at 45 °2θ at 260 °C (Figure 1c and Figure S3). To determine the anion exchange efficiency, we examined both the Se/S ratio measured by SEM-EDS and the extent of the XRD shift, assuming that Vegard’s rule^63^ is applicable here (Tables S1, S2). These two approaches give reasonably similar measurements of the extent of replacement of S by Se and show a continual increase from 7% (150 °C) to 25–29% (180 °C) and 30–36% (260 °C). The TEM images support the idea that the particle shape remained after the ion exchange (Figure 1d, Figure S7). PXRD, TEM, and SEM-EDS data support that didodecyl diselenide does work as a selenium anion post-transformation selenium precursor and the rate of incorporation increases with temperature.

#### Postsynthetic Transformation by Diphenyl Diselenide (PhSe)_2_

Diphenyl diselenide does not induce anion exchange, disproving our initial hypothesis and demonstrating that diorganyl diselenides have different reactivity trends in PST versus direct synthesis; this implies a different interplay between molecular components. (PhSe_)2_ exposure caused dissolution of Cu_2–*x*_S nanorods and reprecipitation of thermodynamically preferred berzelianite Cu_2–*x*_Se nanoparticles, as supported by PXRD and TEM (Figure 1e,f), though it required a higher temperature than for (BzSe)_2_. At 100 and 150 °C, Cu_2–*x*_S nanorods exposed to (PhSe)_2_ maintained the original particle morphology according to TEM (Figure 1f) and showed little Se incorporation (Se/S < 0.3, Table S1). At 100 °C, the crystalline phase was unchanged from the roxbyite Cu_2–*x*_S phase before the exposure, while a mixture between roxbyite Cu_2–*x*_S and berzelianite Cu_2–*x*_Se (peaks at 45 and 37° 2θ) is observed at 150 °C (Figure 1e). It was not until 180 °C that the TEM shows a change in particle shape (Figure 1f) accompanied by an increase in Se/S ratio to 1.4 ± 0.1 (Table S1). Some nanorods remain, although they appear to be partially dissolved to give a greater variety of smaller nanoparticles (Figure 1f). Large particles have also formed (Figure 1f), indicating the reprecipitation of Cu_2–*x*_Se from solution. At 260 °C, Cu_2–*x*_S nanorods largely dissolved and little material reprecipitated, which limited our ability to obtain PXRD. TEM The of the few particles that centrifuged out of the solution shows small, amorphous particles characteristic of rapid reprecipitation. While dissolution and reprecipitation of Cu_2–*x*_S nanoparticles by (PhSe)_2_ did require higher temperatures than that observed for (BzSe)_2_, which is consistent with the stronger C–Se bonds, Se^2–^ anion exchange was not observed.

#### Rationalizing Reactivity Trends with Thermal Decomposition

To explain the PST-specific reactivity trend we found and identify why only (C_12_Se)_2_ of the three diselenides examined induced Se^2–^ anion exchange, we sought to better understand the particular thermal decomposition products formed by all three diselenide species. Rationalization of the PST behaviors of these diorganyl diselenides was informed by computational modeling of solution species using the ReaxFF potential, illustrating a new application for this tool in the mechanistic understanding of rational design of nanoparticles. Such force-field-based molecular dynamics can atomistically simulate large systems and longer times with lower computational power than DFT and as such is a promising exploratory approach to develop a new hypothesis. The ReaxFF potential^34–36^ in particular is a computational framework designed to simulate chemical reactivity and that has been used for modeling metal chalcogenide systems^64–67^ as well as methyl thiolate interactions with copper surfaces.^68^ Due to this accessibility, applicability to similar systems, and low computational demands, we started our investigation with comparing the theoretical thermal decomposition of (C_12_Se)_2_, (PhSe)_2_, and (BzSe)_2_ using molecular dynamics simulations with a newly trained ReaxFF force field. Building off the ReaxFF force field developed for Cu, S, C, and H by Yeon et al.,^68^ a new force field was developed by adding Se parameters^67^ (Supporting Information). The results were vetted by comparing relative bond strengths with DFT (Figures S8–S12 and Table S3).^46^ Having established that the ReaxFF force field calculates appropriate relative bond strengths, we used MD calculations (Figure 2, Figure S13) to model the thermal decomposition pathways of (PhSe)_2_, (BzSe)_2_, and (C12Se)_2_ in the hopes that this would identify differences that could help explain a reactivity trend in which only (C_12_Se)_2_ promoted Se^2–^ anion exchange.

**Figure 2:**
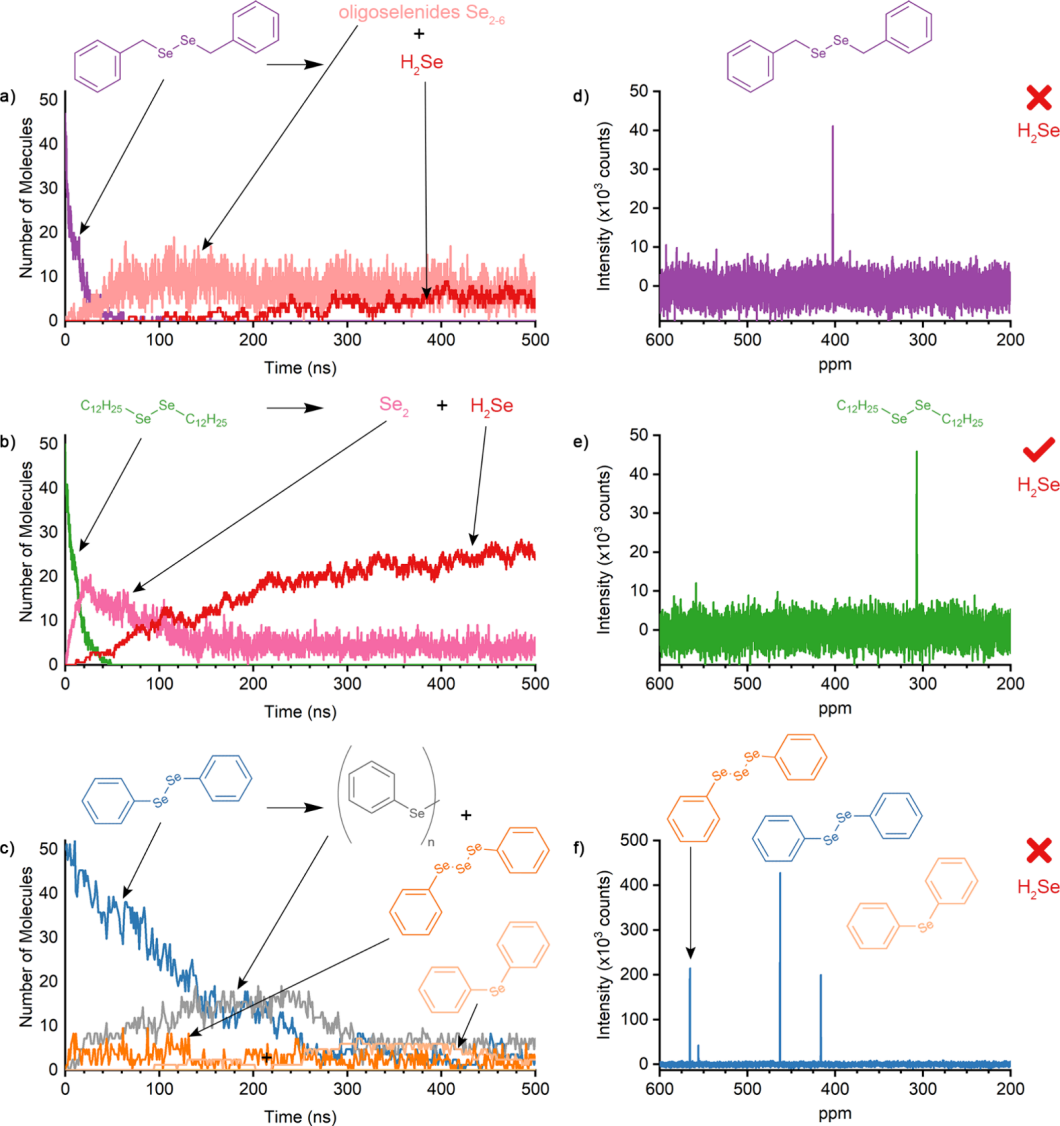
Molecular dynamics simulations (a–c) and experimental results (d–f) of the thermal decomposition of (BzSe)_2_ (purple, top), (C_12_Se)_2_ (green, center), and (PhSe)_2_ (blue, bottom). ReaxFF nVT molecular dynamics simulations were performed on densified ensembles of 50 molecules of interest at 2000 K, and the most frequent Se-containing products are shown. (a–c) MD simulations show different decomposition pathways for the different diselenides, notably the extent of generation of H_2_Se. Chemical structures of each species or collection of species are shown. Experimental thermal decomposition data at 260 °C (d–f) show ^77^Se NMR and whether H_2_Se was formed as indicated by lead acetate paper. (d) Postdecomposition ^77^Se NMR of (BzSe)_2_ only shows (BzSe)_2_ (403 ppm), while BzSeBz (331 ppm) and Bz_2_Se_3_ (538 and 410 ppm)^39^ are notably absent. (e) Postdecomposition ^77^Se NMR of (C12Se)_2_ similarly shows only a single peak in 307 ppm corresponding to (C_12_Se)_2_, and formation of H_2_Se is observed using lead acetate tape. (f) Postdecomposition ^77^Se NMR of (PhSe)_2_ results in several products including phenyl triselenide (560 and 470 ppm), phenyl monoselenide (420 ppm), and PhSe_3_ (tentatively attributed to the small peak at 555 ppm). PhSe (−10 ppm) is predicted but not observed, despite reports of its formation at 200 and 220 °C.^39^

Comparison of the thermal decomposition MD simulations of (BzSe)_2_, (PhSe)_2_, and (C12Se)_2_ gave rise to a new hypothesis that the release of H_2_Se is the key driver of Se^2–^ anion exchange. Based on these simulations, H_2_Se was the primary Se-containing product formed upon heating (C12Se)_2_ but not when heating (BzSe)_2_ or (PhSe)_2_, which suggested that the ability to form *in situ* H_2_Se may differentiate whether a particular diorganyl diselenide drives Se^2–^ anion exchange (Figure 2a–c). At 1000 and 1500 K, all three diselenides primarily oligomerize by forming Se–Se bonds and do not show bond-breaking (Figure S13, Table S4). We started simulations at low temperatures, but after observing little reaction at 1000 and 1500 K, we selected 2000 K as the focus for thermal decomposition MD simulation. Increasing the simulation temperature to 2000 K revealed distinct chemical decomposition products that make sense given the relative bond strengths (Table S3 and Figure S12). (BzSe)_2_ primarily breaks down into oligoselenides through cleavage of the weak C–Se bond and formation of new Se–Se bonds. Small amounts of H_2_Se are observed concurrent with oligoselenides starting at 200 ns and rising to include ∼5% of the total Se atoms (Figure 2a). (C_12_Se)_2_ initially breaks down into Se_2_, but then, H_2_Se starts forming and grows to include ∼30% of the total Se atoms (Figure 2b). This breaking of both the C–Se and Se–Se bonds reflects the relative similarity of the bond strengths in (C_12_Se)_2_. The lower C–H bond strength for sp^3^ C allows for protonation of Se (Figure S14). (PhSe)_2_ is shown to form numerous species that retain the strong C–Se bond, which we classified as oligomers (species with new Se–Se bonds that retain the C–Se bonds), phenyl triselenides (various species that have 3 Se and connected Ph groups), and phenyl monoselenide (PhSe) (Figure 2c). Of the decomposition products formed at 2000 K, H_2_Se gas has already been used to selenize metal sulfides to make BaZr­(S,Se)_3_ ^30^ and Cu­(Ga,In)­(S,Se)_2_ ^69^ and is thus most likely to be causing selenization here. To ensure the validity of the MD simulations, we sought to compare the results to experimental thermal decomposition at the temperatures used here (260 °C).

#### Validation of MD Simulations by Diorganyl Diselenide Thermal Degradation

The thermal decomposition reactions validated the MD simulations and supported our hypothesis that H_2_Se production, identified by MD, is a predictor that an organyl selenide species could drive selenization by anion exchange. We sought to confirm this prediction by thermal decomposition of (C_12_Se)_2_ at temperatures similar to those used for Se^2–^ anion exchange (Figure 2d–f, Figures S15, S16) and directly comparable to published decomposition studies of (BzSe)_2_ or (PhSe)_2_.^39^ Following the procedure from Koziel et al.,^39^ samples (BzSe)_2_, (PhSe)_2_, and (C12Se)_2_ were heated at 260 °C for 120 min. Note that neither 1-octadecene and oleylamine, despite being present during the reaction, were added to avoid convoluting thermal decomposition with additional known reactions.^39^ The thermal decomposition was assessed through ^77^Se NMR, and the presence of H_2_Se gas was detected using lead acetate tape and the color-change of the white rubber septa (Figures S15 and S16).

The decomposition of (BzSe)_2_ resulted in a single peak in the ^77^Se NMR spectrum, which aligns with the undecomposed sample of (BzSe)_2_ (Figure 2d). Black solid precipitated at the bottom of the NMR tube, previously identified as metallic Se.^39^ The decreased ^77^Se NMR signal observed for (BzSe)_2_ is likely due to the formation of this metallic Se. While MD predicted low levels of H_2_Se formation, no color change of the lead acetate tape was observed, consistent with reports.^39^ Lower temperature decomposition analysis has been reported to yield also the monoselenide BzSeBz and Bz_2_Se_3_,^39^ but neither species was observable here. The MD simulations at 1500 K (Figure S13) agreed with the lower temperature decomposition in that Bz_2_Se_3_ and BzSeBz were both common products as well as numerous other oligoalkyl selenides in roughly equivalent amounts. The MD simulation at 2000 K, however, showed that (BzSe)_2_ primarily decomposed into oligoselenides, with Se_3_, HSe_4_, Se_4_, and HSe as the most common products. Se_2_ does not show a ^77^Se NMR signal, while HSe^–^ would appear at −496 ppm but is not observed here (see Figure S15 for an expanded view of the NMR spectra).^70^ Though these were not observed in NMR, such reactive species could reasonably contribute to the formation of the observed solid Se.

Thermal decomposition of (C12Se)_2_ did not result in any new signals in the ^77^Se NMR (Figure 2e), but the lead acetate tape changed to a reddish color, confirming the release of H_2_Se (Figure S16). The presence of Se on the lead acetate tape was validated by EDS. The intensity of the ^77^Se NMR signal is reduced compared to (PhSe)_2_ despite the same initial concentration, indicating a loss of Se-species. The rubber septum reacted with the headspace in the NMR tube to turn black (Figure S16). All of this evidence supports the generation of H_2_Se, in agreement with the MD simulation.

Thermal decomposition of (PhSe)_2_ resulted in phenyl triselenide and phenyl monoselenide, in agreement with published reports and the MD simulations (Figure 2f). The products predicted by MD were slightly more varied, but the four most commonly formed Se-containing molecules were PhSe_3_, PhSe_3_Ph, PhSePh, and PhSe (not observed here but reported at 200 and 220 °C) (Table S4). The greater intensity of the ^77^Se NMR spectra speaks to the greater stability of (PhSe)_2_, in that little Se was lost to Se(0) or H_2_Se. Indeed, no evidence of H_2_Se generation was observed based on the absence of color change of lead acetate tape (Figure S16).

### Rational Design of New Anion Exchange Promoters

The identification of H_2_Se generation by MD with our new ReaxFF force field as a rationale for Se^2–^ anion exchange by (C_12_Se)_2_ led us to ask whether MD could be used to identify Se^2–^ anion exchange reagents *a priori*. We extrapolated from our rationale that (C_12_Se)_2_ worked as a selenization agent due to the similar C–Se and Se–Se bond strengths and numerous sp^3^ C–H bonds that can protonate Se and create H_2_Se. We thought that dialkyl monoselenides might similarly produce H_2_Se. Monoselenides have not been as widely used as diselenides for direct synthesis of nanomaterials due to their greater instability. Monoselenides have, however, shown a propensity to bind to metal centers as neutral ligands, allowing for construction of single-source coordination complexes^54^ applied as precursors to Cu_2_Se^71^ and AgCuSe nanoparticles.^72^ Thus, we used ReaxFF to simulate thermal decomposition of two related species, didecyl diselenide (C_10_Se)_2_ and didecyl monoselenide, (C_10_)_2_Se (Figure 3a, Table S5). Due to the chemical similarities with (C_12_Se)_2_, both (C_10_Se)_2_ and (C_10_)_2_Se produced H_2_Se as the primary Se-containing product. The change in alkyl chain length was not found to alter its selenizing ability, in keeping with the similarity in C–Se and Se–Se bond strengths for dialkyl diselenides with long alkyl chains with even numbers of carbon (Table S6).

**Figure 3:**
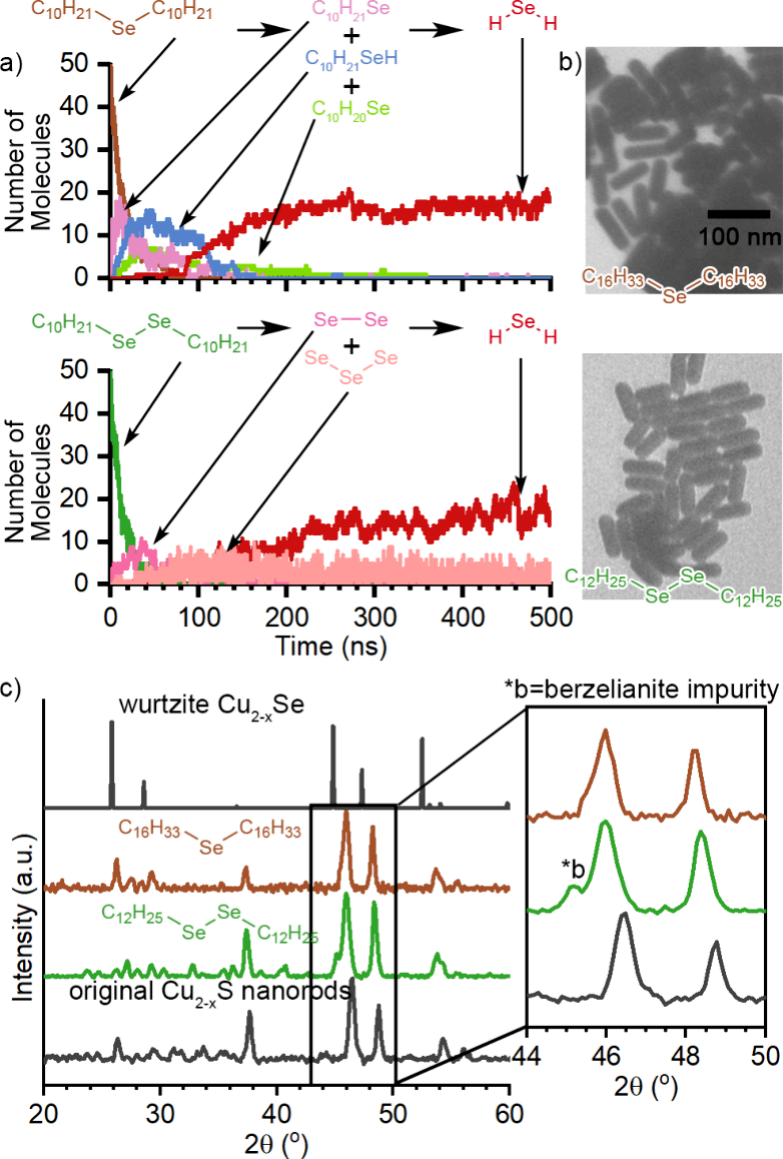
Comparison of Se^2–^ anion exchange with dialkyl diselenides versus dialkyl monoselenides. (a) MD simulations of (C_10_)_2_Se (top) and (C_10_Se)_2_ (bottom) at 2000 K show that both species produce H_2_Se and therefore are predicted to promote anion exchange. (b) TEM images postanion exchange show retention of the nanorod morphology for both (C_16_)_2_Se (top) and (C_12_Se)_2_ (bottom). (c) PXRD comparing the particles before and after exchange to the wurtzite Cu_2–*x*_Se pattern shows that they both retain hcp but that (C_16_)_2_Se has a slightly greater shift, indicating more Se incorporation and lacking a berzelianite phase impurity.

Comparing the mono- and diselenides, (C_10_)_2_Se forms H_2_Se faster; while (C_10_)_2_Se has decomposed into 20 molecules of H_2_Se by 200 ns, (C_10_Se)_2_ has <10 molecules. The identity of the Se-species formed before H_2_Se also changes with the mono- versus diselenides. Because of the pre-existing Se–Se bonds, (C_10_Se)_2_ forms the oligoselenides Se_2_ and Se_3_, while (C_10_)_2_Se generates C_10_H_20_Se, C_10_H_21_Se, and C_10_H_21_SeH. Gahlot et al. have also noted that this cleaner decomposition makes the monoselenides an interesting alternative chalcogenide source for metal chalcogenide synthesis.^72^ Having confirmed our proposal that dialkyl monoselenides would generate H_2_Se using MD simulation, we then synthesized (C_16_)_2_Se (assuming based on the calculations that the alkyl chain length was not crucial) and attempted anion exchange at 260 °C (Figure 3). Indeed, Se incorporation was observed (Se/S mole ratio of 0.47 ± 0.04, an anion exchange efficiency of 25%), and the nanorod morphology was retained (Figure 3b), as was the hcp wurtzite structure (Figure 3c). Notably, the shift to lower 2θ due to incorporation of Se was slightly greater than that for (C_12_Se)_2_. Furthermore, greater phase purity was observed using (C_16_)_2_Se compared to (C_12_Se)_2_. A small berzelianite impurity is present (note the shoulder at 45 °2θ) after anion exchange with the diselenide, perhaps due to dissolution and reprecipitation of Cu_2–*x*_Se by the oligoselenides from which H_2_Se formed in dialkyl diselenides (Figure 2b). In comparison, the alkyl monoselenides produced during the initial decomposition of the monoselenide should not easily dissolve the copper sulfide. Low-reactivity side-products for dialkyl monoselenides may contribute to their being superior Se^2–^ anion exchange reagents. Dialkyl monochalcogenides have been used for MOCVD reactions,^73^ and in the direct synthesis of iron sulfides, though some diorganyl sulfides failed to grow particles.^13^ These initial findings reveal that the behavior of dialkyl monochalcogenides is another intriguing comparison point between direct growth versus PST.

## Conclusions

By examining diorganyl diselenide reactivity trends, we identified the molecular driving forces for exchanging S^2–^ anions for Se^2–^ in Cu_2–*x*_S nanorods employing diorganyl diselenides as the selenium source. Unlike in direct growth where the relative C–Se versus Se–Se bond strength is a crucial deciding factor determining the crystalline phase, we found that the saturation of the diorganyl diselenides determined whether a species induced anion exchange. By coupling molecular dynamics calculations using the ReaxFF approach with our experimental findings, we developed a new hypothesis wherein anion exchange is driven by the slow release of H_2_Se due to thermal decomposition of the diselenide. The (alkylSe)_2_ species released H_2_Se because of the combined similarity of their C–Se and Se–Se bond strengths and facile C–H bond cleavage. This hypothesis was supported by the thermal decomposition of these diorganyl diselenides via molecular dynamics simulations and molecular degradation experiments evaluated by ^77^Se NMR and detection of H_2_Se. Having demonstrated the utility of modeling thermal decomposition using accessible, low-computational-power molecular dynamics simulations with ReaxFF to predict conditions under which selenization will occur, we used this tool to identify a new Se^2–^ anion exchange reagent, dialkyl selenide.

Rational design of the nanoparticle phase, shape, and composition remains a challenging goal, but this work provides several new tools to achieve it. Not only do we have new selenization agents and a design scheme for discovering more, but we have also revealed a safer source of H_2_Se­(g) that can be employed in other ways without requiring cylinders of this toxic gas. ReaxFF MD simulations, with appropriately trained force field parameters, can be used to gain molecular-level insights into nanoparticle growth and transformation processes, including *a priori* prediction of reagents for postsynthetic transformation. We delivered a new tool to comprehend a complex reaction mechanism by exploring species used in both direct synthesis and PST contexts and showed it to bear useful insights into both processes. While direct synthesis and PST have orthogonal reactivity trends, diorganyl diselenides and diorganyl monoselenides have unique uses in both venues and a potential for being tailored to provide better synthetic control by manipulating the molecule’s functionality based on temperature setting, forming new nanoheteromaterials. Continuing to search for parallels and orthogonalities between these two processes will improve the community’s ability to design new nanostructures by application of consecutive synthetic tools, analogous to the process of organic total synthesis. We hope the diligent work that has been undertaken to bring detailed understanding of the synthesis of phase-, size-, and composition-controlled nanoparticles can be extended even more broadly to other postsynthetic transformations, thus creating highly controlled synthetic systems to enable synthesis of new, complicated, multicomponent nanoheterostructures tailored to specific functions.

## Supplementary Material







## Data Availability

Raw data used to make Figures 1–3 including PXRD, TEM, and EDS characterization of samples, the xyz files showing the MD simulations, and NMR spectra after thermal decomposition can be found at 10.17605/OSF.IO/GRZ9Q.

## Notes

Information on detailed author roles according to the CRediT system and additional experimental and computational data (Figures S1–S16 and Tables S1–S6) Initial Cartesian coordinates (xyz) for MD simulations ReaxFF force field (PDF)
Initial Cartesian coordinates for MD simulations (TXT)
ReaxFF force field (XYZ)

## ABBREVIATIONS

(C_12_Se)_2_: diselenide
(PhSe)_2_: diphenyl diselenide
(BzSe)_2_: dibenzyl diselenide
(C_10_Se)_2_: didecyl diselenide
(C_10_)_2_Se: didecyl selenide
(C_16_)_2_Se: dihexadecyl selenide
