# Supplementary material for: Copper Selenides via Anion Exchange versus Direct Growth – The Role of Diorganyl Diselenides
Source: Inorg Chem. 2025 Nov 14;64(47):23294–304. doi: 10.1021/acs.inorgchem.5c04328 (PMC12710604; doi:10.1021/acs.inorgchem.5c04328)
Supplement: Supplementary file 1 [file ic5c04328_si_001.pdf]

SUPPORTING INFORMATION

Copper selenides via anion exchange versus direct growth – The role of diorganyl diselenides

Jiwoo Choi, [a†] *Benjamin A. Schmidt,* [a†] *Mykhailo Boleychuk,* [a] *Kiran Bedi,*[a,‡] *Emily Sandoval-Arteaga,*[a, ‡] *Kezia N. Almonte,*[a, ‡] *Quentin M. Boussard,*[a] *J. Kenneth Krebs,*[b] *Malgorzata Kowalik,*[c] *Adri van Duin,*[c] *Katherine E. Plass* [a,*]

[a]Department of Chemistry, Franklin & Marshall College, Lancaster, Pennsylvania 17604, United States

[b]Department of Physics, Franklin & Marshall College, Lancaster, Pennsylvania 17604, United States

[c]Department of Mechanical Engineering, Pennsylvania State University, University Park, Pennsylvania 16802, United States

*kplass@fandm.edu

Table of contents

	Page
**Table S0.** Author contributions according to CRediT Contribution Roles Taxonomy	S3
**Experimental and Computational Details**	S4
**Table S1.** Summary of the mole ratios measured after post-synthetic transformation by $(BzSe)_2$, $(C12Se)_2$, and $(PhSe)_2$ by SEM-EDS	S6
**Discussion of phase assignment after PST with $(C12Se)_2$**	S7
**Figure S1.** Comparison of the reference patterns for wurtzite Cu_2Se, berzelianite Cu_2Se, and roxbyite $Cu_{2-x}S$	S8
**Figure S2.** Comparison of the reference patterns for wurtzite Cu_2Se and roxbyite $Cu_{2-x}S$ and PXRD patterns for Se^{2-} exchanged particles with matched solid-solutions.	S9
**Figure S3.** PXRD pattern for Se^{2-} exchanged particles at 260 °C with matched patterns of contracted $Cu_{2-x}Se$ wurtzite and expanded roxbyite $Cu_{2-x}S$ structures.	S10
**Figure S4.** PXRD pattern for Se^{2-} exchanged particles at 180 °C with matched patterns of contracted $Cu_{2-x}Se$ wurtzite and expanded roxbyite $Cu_{2-x}S$ structures.	S11
**Figure S5.** P PXRD pattern for Se^{2-} exchanged particles at 150 °C with matched patterns of contracted $Cu_{2-x}Se$ wurtzite and expanded roxbyite $Cu_{2-x}S$ structures.	S12
**Figure S6.** PXRD pattern for Se^{2-} exchanged particles at 100 °C with matched patterns of contracted $Cu_{2-x}Se$ wurtzite and expanded roxbyite $Cu_{2-x}S$ structures.	S13
**Table S2.** Analysis of the anion exchange efficiency based on SEM-EDS and XRD assuming Vegard's law.	S14
**Figure S7.** Population analysis of length and width of nanorods after PST with $(C12Se)_2$ demonstrating size and shape retention.	S15
**Figure S8.** DFT and ReaxFF energies for the C-Se bond dissociation in H_3C-SeH.	S15
**Figure S9.** DFT and ReaxFF energies for the H-Se bond dissociation in H_2Se.	S16
**Figure S10.** DFT and ReaxFF energies for the H-Se-H angle distortion in H_2Se.	S16
**Figure S11.** ReaxFF and DFT C-Se bond dissociation energies in diorganyl diselenide molecules.	S17
**Table S3.** C-Se and Se-Se bond strengths and the difference between then in $(BzSe)_2$, $(CH_3Se)_2$, and $(PhSe)_2$ as calculated by the optimized ReaxFF force field and reported from DFT calculations.	S17
**Figure S12.** C-Se and Se-Se bond strengths and the difference between then in $(BzSe)_2$, $(CH_3Se)_2$, and $(PhSe)_2$ as calculated by the optimized ReaxFF force field and reported from DFT calculations.	S18
**Figure 13.** Plot of the Se-containing species formed via MD simulations of thermal decomposition of $(BzSe)_2$, $(C12Se)_2$, and $(PhSe)_2$ at 1000 K, 1500 K, and 2000 K.	S19
**Table S4.** Listing of the species formed via MD simulations of thermal decomposition of $(BzSe)_2$, $(C12Se)_2$, and $(PhSe)_2$ at 1000 K, 1500 K, and 2000 K.	S20
**Figure 14.** C-H bond strengths in $(BzSe)_2$, $(C12Se)_2$, and $(PhSe)_2$ as calculated by the optimized ReaxFF force field.	S21
**Figure 15.** Additional data on thermal decomposition of $(BzSe)_2$, $(C12Se)_2$, and $(PhSe)_2$ at 260 °C, including an expanded view of ^{77}Se NMR.	S22
**Figure 16.** Pictures of the lead acetate tape and rubber septa exposed to the head space during thermal decomposition of $(BzSe)_2$, $(C12Se)_2$, and $(PhSe)_2$ at 260 °C.	S23
**Table S5.** Listing of the species formed via MD simulations of thermal decomposition of $(C10Se)_2$ and $(C10)_2Se$ at 1000 K, 1500 K, and 2000 K.	S24

**Table S6.** Bond dissociation energies for even long-chain dialkyl diselenides calculated with ReaxFF.	S25
**References**	S25

Table S0. Author contributions according to CRediT Contribution Roles Taxonomy

Author	Contribution	Detail
Jiwoo Choi – co-1st author	Investigation (lead), Visualization, Writing – Original Draft, Writing – Review & Editing.	Led experiments to carry out post-synthetic transformations of $Cu_{2-x}S$ nanorods with various diorganyl diselenides and synthesized $(C12Se)_2$ (Figure 1d-f, S1); carried out thermal decomposition experiments (Figure 2c, S5); led creation and design of Figures 1 and 2c; contributed to early drafts of the manuscript; contributed to reviewing and revising the manuscript and figures.
Benjamin Schmidt – co-1st author	Investigation (lead), Visualization, Writing – Original Draft, Writing – Review & Editing.	Developed approach and carried out MD simulations (Figures 2ab, 3a, S2); vetted force field through bond-strength and PES scans (Figures S3, S4); led creation and design of Figures 2a,b, S2, S3, S4; contributed to early drafts of the manuscript; contributed to reviewing and revising the manuscript and figures.
Mykhailo Boleychuk – 2nd author	Investigation (lead), Writing – Review & Editing.	Synthesized $(C16)_2Se$ and carried out post-synthetic transformation of $Cu_{2-x}S$ nanorods with it (Figure 3); contributed to reviewing and revising the manuscript and figures.
Kezia Almonte – co-3rd author	Investigation (supporting), Writing – Original Draft, Writing – Review & Editing.	Carried out MD and bond-strength calculations to vet force field; contributed to reviewing and revising the manuscript and figures.
Emily Sandoval-Arteaga – co-3rd author	Investigation (supporting), Writing – Review & Editing.	Carried out post-synthetic transformations of $Cu_{2-x}S$ nanorods with various diorganyl diselenides (Figure 1)
Kiran Bedi – co-3rd author	Investigation (supporting), Writing – Original Draft, Writing – Review & Editing.	Carried out post-synthetic transformations of $Cu_{2-x}S$ nanorods with various diorganyl diselenides (Figure 1); contributed to drafts of the manuscript.
Quentin M. Boussard – 4th author	Investigation (supporting)	Developed synthesis of $(C12)_2Se$.
J. Kenneth Krebs	Supervision, Conceptualization	Co-lead the nanobots Student Research Club that helped validate the force field and explored various approaches to modeling the behavior of diorganyl selenides.

Malgorzata Kowalik	Conceptualization, Methodology, Writing – Review & Editing.	Helped students learn to do bond strength calculations and MD simulations with ReaxFF in the AMS software suite. Guided calculation design.
Adri van Duin	Conceptualization, Methodology, Writing – Review & Editing.	Developed ReaxFF force field including several iterations.
Katherine E. Plass	Conceptualization, Resources, Writing – Original Draft, Supervision, Project administration, Funding acquisition	Initiated project, oversaw student work, and acquired funding. Wrote manuscript and revised figures from student drafts and original text and data analysis.

ADDITIONAL EXPERIMENTAL AND COMPUTATIONAL DETAILS

Characterization

Powder X-Ray Diffraction (PXRD). After nanoparticles were cleaned and resuspended in heptane, they were cast onto glass slides and allowed to dry. The PXRD data were collected using a PANalytical X'Pert Pro X-ray diffractometer with Cu Ka radiation. The samples were scanned with 10 repetitions at a current of 40 mA and voltage of 45 kV. Using the PANalytical HighScore Plus software, the ten scans were summed and were compared patterns from the ICDD database to determine the structure of the nanoparticles. Crystal structure and powder diffraction simulations were performed using CrystalMaker and CrystalDiffract from CrystalMaker Software Ltd., Oxford, England.

Transmission Electron Microscopy (TEM). Samples were prepared by placing a drop (from a glass pipette) of nanoparticles suspended in toluene on a Au-supported ultra-thin carbon-coated TEM grid (Electron Microscopy Sciences). TEM images of the particles and their average sizes were obtained using a Delong Instruments LVEM25 Low-Voltage TEM at Franklin & Marshall College. The LVEM25 was operated under 25 kV with the Zyla 5.5 Scientific CMOS camera with appropriate alignments and enhancements. ImageJ software was used to analyze and measure the particles in the TEM images.

Scanning electron microscopy/energy-dispersive X-ray spectroscopy (SEM/EDS). Nanoparticles previously cast onto the PXRD slides were immobilized on a small piece of conductive carbon tape and affixed to a metal stub. SEM and EDS of the sample were then carried out at 20 kV with the Evex Mini-SEM. Atomic ratios were determined at 5 spots or more per sample to determine the averages and standard deviations.

NMR ^{1}H and ^{77}Se NMR Spectra were obtained on a Bruker AVANCE NanoBay 400 MHz spectrometer. Chemical shifts (^{1}H and ^{77}Se) are reported in parts per million and referenced to the solvent peak (for $CHCl_3$, δ = 7.26 ppm).

Computational modeling

Force field vetting and bond strength comparison

Simulations were performed using the Amersterdam Modelling Suite (AMS 2023 or AMS 2024)[1,2] on with ReaxFF (laptops) or DFT (the Franklin and Marshall College Cluster). The data obtained based on the newly developed ReaxFF parameter set (see SI) were compared to the DFT data obtained with use of the hybrid exchanged-correlation functionals B3LYP-D3 with the TZP basis set. The initially build diorganyl diselenide molecules were geometry optimized (using the following stopping criteria: 0.001 Ha/Å change for gradient, 0.00001 Ha change for energy or 0.01 Å step for change in the position) and the energy of these minimized structures were recorded as a baseline. To assess a bond dissociation energy, for C-Se and Se-Se bonds, a distance between the bonded atoms was increased to 3000 pm and the energies of these structures with the broken bonds were compared to the initially minimized ones. The DFT data were further treated as a target in the optimizing ReaxFF parameter set. Also, the potential energy surface (PES) scans were utilized in optimizing the ReaxFF parameters set for the considered diorganyl diselenides.[3] In addition, the DFT calculations allowing for calculations of the bond dissociations for the C-Se and H-Se in H_3C-SeH and H_2Se, respectively, were performed as well as on H_2Se to determine the

H-Se-H angle distribution and all this information were also incorporated in the ReaxFF parameter set optimization process. The parameters for the Se-Se interaction were taken from previous ReaxFF work on SnSe-material synthesis.[4] Figures S13-S15 compare the DFT and ReaxFF results for these bond dissociation and angle distortion curves. Figures S16 and S17 show the ReaxFF and DFT C-Se and Se-Se bond dissociation energies in various diorganic diselenide compounds and demonstrate that ReaxFF reproduces the bond dissociation trends as obtained from the DFT calculations. We focused on accurately simulating the C-Se and Se-Se bond strengths to generate diorganyl diselenide (R-Se-Se-R). The bond dissociation data from the ReaxFF calculations for various diorganyl diselenides aligned with the literature data[5,6] and reproduced the differences in the relative bond strengths for $(BzSe)_2$, $(CH_3Se)_2$, and $(PhSe)_2$. Upon comparison of C-Se and Se-Se bond strength (DBDE), $(PhSe)_2$ has a distinctly stronger C-Se bond (DBDE = 18.2 kcal/mol ReaxFF vs literature 20.9-22.55, Table S3) while $(BzSe)_2$ has a much stronger Se-Se bond (DBDE = -7.11 kcal/mol with ReaxFF vs -10.22 kcal/mol literature, Table S3). Dimethyl diselenide has quite similar C-Se and Se-Se bond strengths, leading to a small (DBDE = 4.87 kcal/mol ReaxFF vs. 1.82 and 3.12 kcal/mol literature, Table S3).

Temperature of PST	$(BzSe)_2$		$(C12Se)_2$		$(PhSe)_2$	
	Se/S mole ratio	Cu/(S+Se) mole ratio	Se/S mole ratio	Cu/(S+Se) mole ratio	Se/S mole ratio	Cu/(S+Se) mole ratio
260 °C	12±1	0.7±0.2	0.60±0.07	0.91±0.09	-	-
180 °C	0.08±0.04	1.5±0.2	0.5±0.2	0.9±0.2	1.4±0.1	1.0±0.2
150 °C	1.3±0.4	0.99±0.07	0.13±0.01	1.7±0.1	0.29±0.03	1.3±0.2
100 °C	0.07±0.01	1.8±0.3	0.10±0.04	1.35±0.03	0.14±0.12	1.5±0.3

Table S1. Summary of the mole ratios measured after post-synthetic transformation by $(BzSe)_2$, $(C12Se)_2$, and $(PhSe)_2$ by SEM-EDS. Averages and standard deviations come from measuring 5 different areas.

Discussion of phase assignment after PST with $(C12Se)_2$

Post-synthetic transformation of $Cu_{2-x}S$ with $(C12Se)_2$ results in a hexagonally close-packed $Cu_{2-x}(S,Se)$ solid-solution with the $Cu_{2-x}Se$ wurtzite structure, though some structural features of the initial roxbyite $Cu_{2-x}S$ structure are retained. Comparing these two structures, we see a similarity in the hexagonal close-packing of the chalcogenide sublattice, but differences in the cation ordering (Figure S1). In addition to the tetrahedrally coordinated Cu^+ ions found in wurtzite, there are trigonally coordinated Cu^+ sites in roxbyite. The relatively ordered wurtzite $Cu_{2-x}Se$ structure has 5 primary reflection peaks at 25.8, 28.6, 44.8, 47.3, and 52.5 °2θ and one quite small peak at 36.7 °2θ (Figure S1).[7] The $Cu_{2-x}S$ roxbyite structure (ICSD 185807)[8] is more complicated due to a quasi-hexagonal close-packing of the S (which contributes to the primary reflections at 46.9 and 48.9) and cation ordering that results in numerous small reflections between 24 and 45 °2θ (Figure S1)

Comparison of the pure $Cu_{2-x}Se$ wurtzite and $Cu_{2-x}S$ roxbyite XRD reference patterns versus the patterns obtained after post-synthetic transformation of $Cu_{2-x}S$ with $(C12Se)_2$ (Figure S2) shows the solid-solution behavior. The most prominent peaks between 45 and 49 °2θ continuously shift to lower 2θ values as Se ions increase the lattice spacing between the close-packed anion planes. We compared the PXRD after PST with $Cu_{2-x}S$ with $(C12Se)_2$ at 260 °C (Figure S3), 180 °C (Figure S4), 150 °C (Figure S5), and 100 °C (Figure S6) with both a lattice-contracted wurtzite phase and a lattice-expanded roxbyite phase. We generally found that the spacing between the two highest intensity peaks around 45 and 48 °2θ were easier to match with the wurtzite structure and that the peak around 54 °2θ was matched with either. The peak around 37 °2θ was more prominent in the expanded roxbyite structures but is present in the contracted wurtzite structures. Similar amplification of this peak was also seen in hexagonal $Cu_{2-x}(S,Se)$ nanoparticles.[9] The pattern from 260 °C (Figure S3) and 150 °C (Figure S5) do show some low-intensity peaks that might indicate roxbyite-like cation ordering. Ultimately, the actual structure might be a hybrid

of the wurtzite and roxbyite structures, but clearly retains the hexagonally close-packed anion sublattice expected for a topotactic anion exchange.

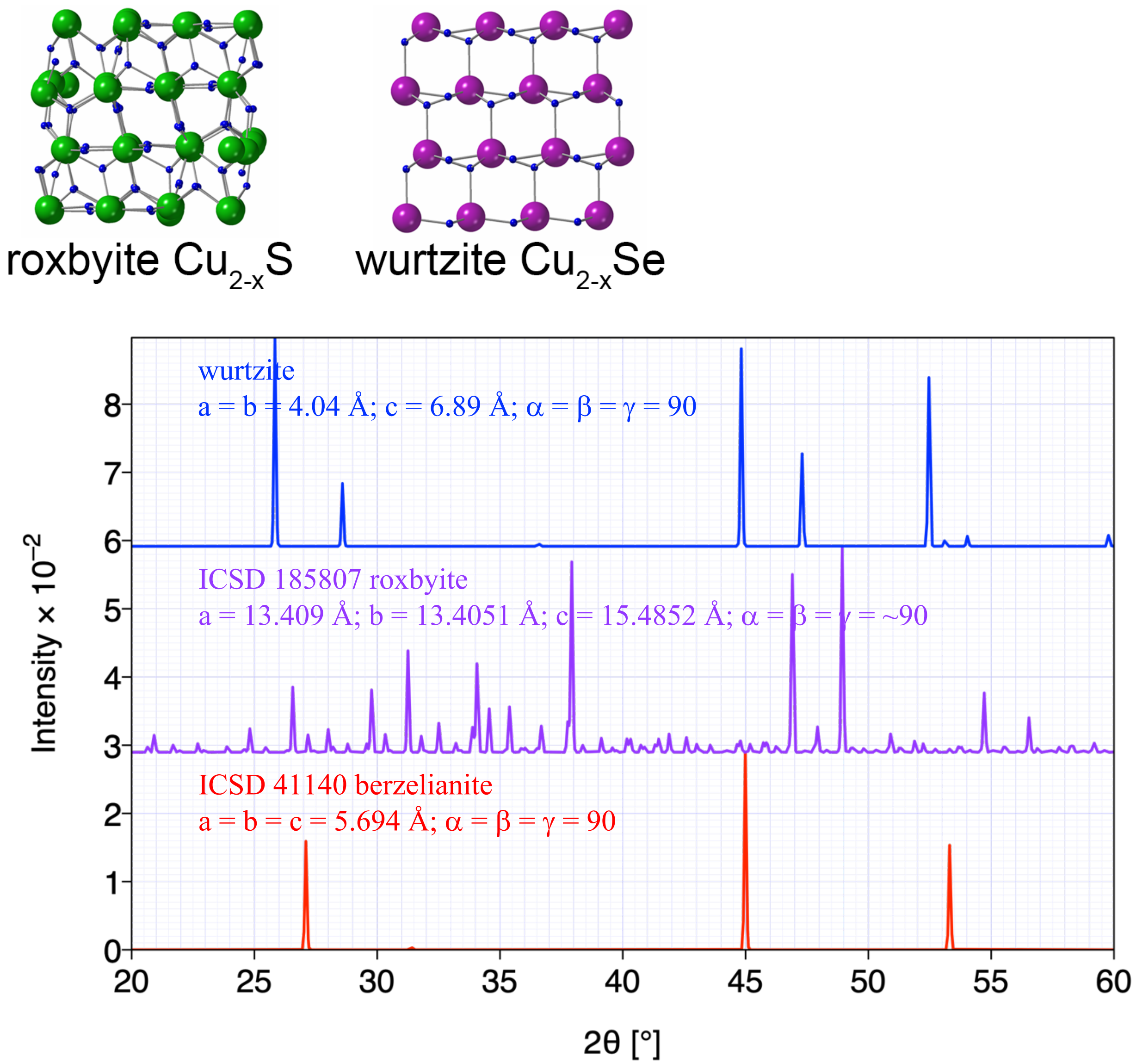

Figure S1. (top) Crystal structures of roxbyite $Cu_{2-x}S$[8] and wurtzite Cu_2Se.[7] (bottom) Comparison of the PXRD reference patterns for wurtzite Cu_2Se,[7] berzelianite Cu_2Se,[10] and roxbyite $Cu_{2-x}S$.[8]

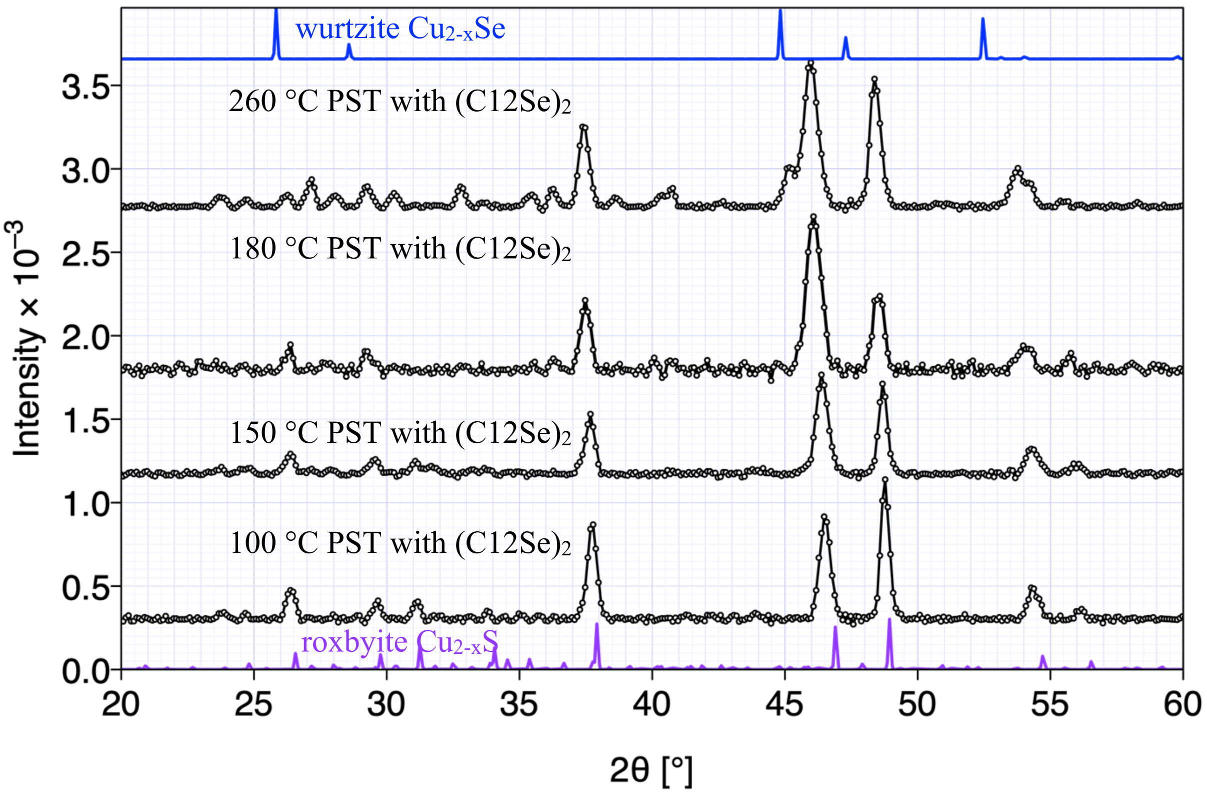

Figure S2. Comparison of the reference patterns for wurtzite Cu_2Se and roxbyite $Cu_{2-x}S$ and PXRD patterns for Se^{2-} exchanged particles with matched solid-solutions.

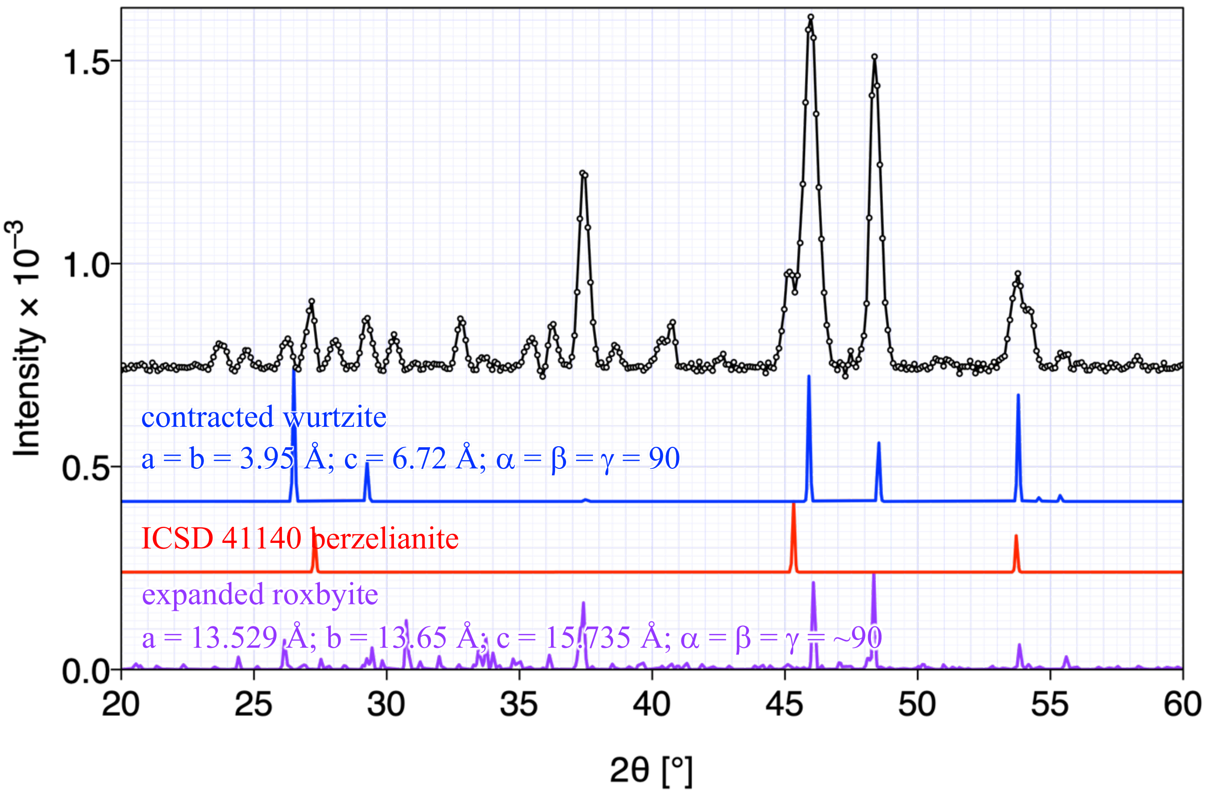

Figure S3. PXRD pattern for Se^{2-} exchanged particles at 260 °C with matched patterns of contracted $Cu_{2-x}Se$ wurtzite and expanded roxbyite $Cu_{2-x}S$ structures as well as berzelianite to show the impurity phase.

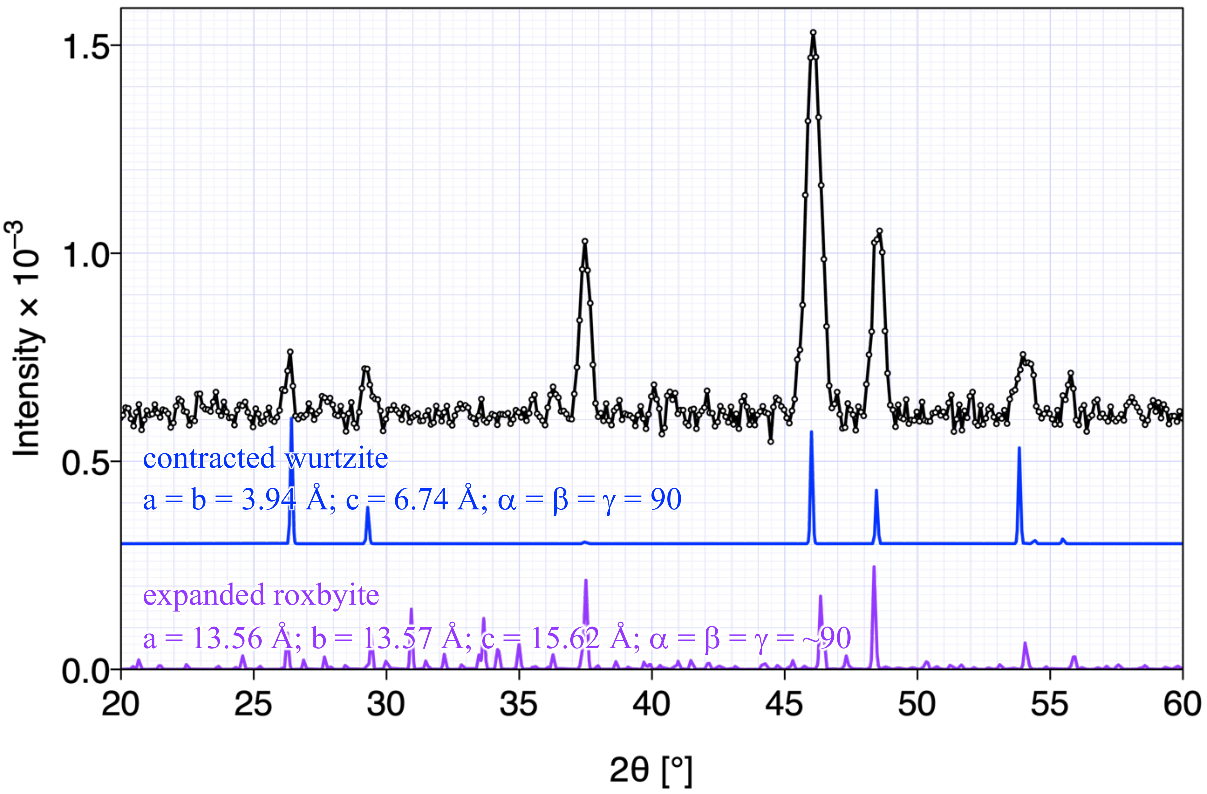

Figure S4. PXRD pattern for Se^{2-} exchanged particles at 180 °C with matched patterns of contracted $Cu_{2-x}Se$ wurtzite and expanded roxbyite $Cu_{2-x}S$ structures.

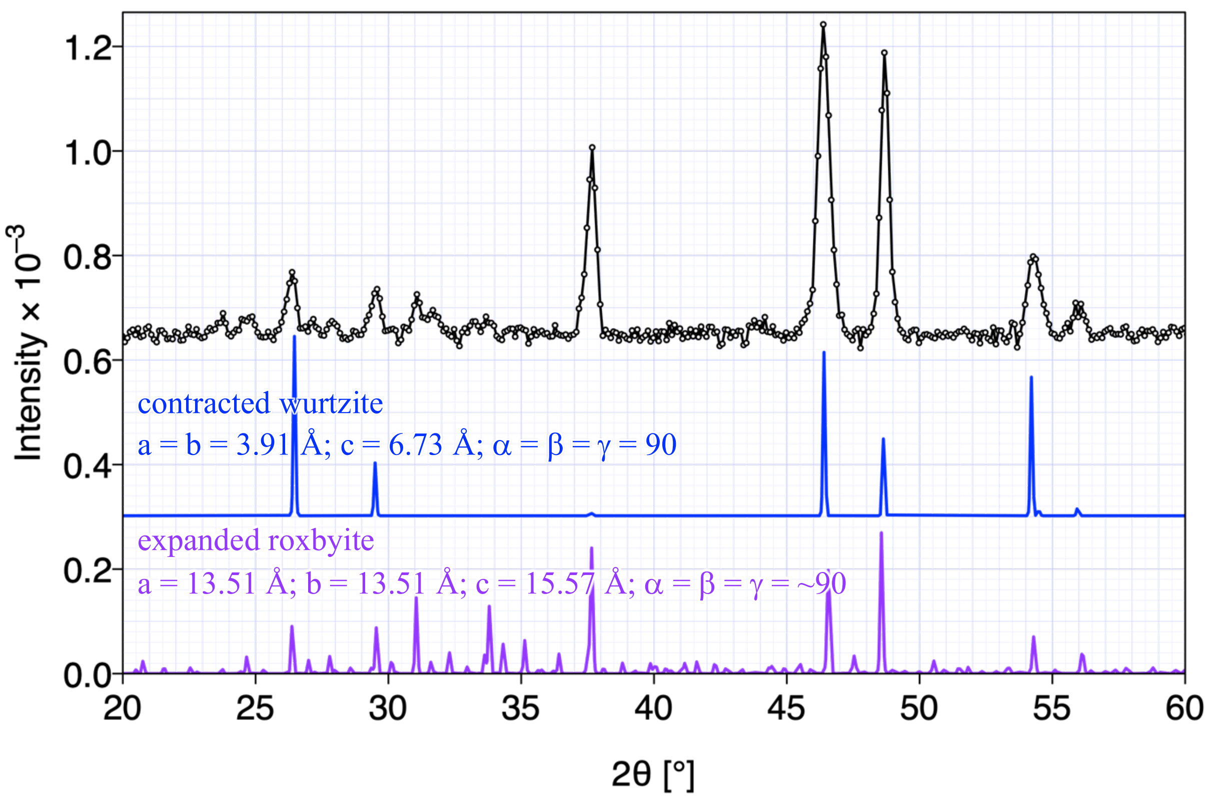

Figure S5. PXRD pattern for Se^{2-} exchanged particles at 150 °C with matched patterns of contracted $Cu_{2-x}Se$ wurtzite and expanded roxbyite $Cu_{2-x}S$ structures.

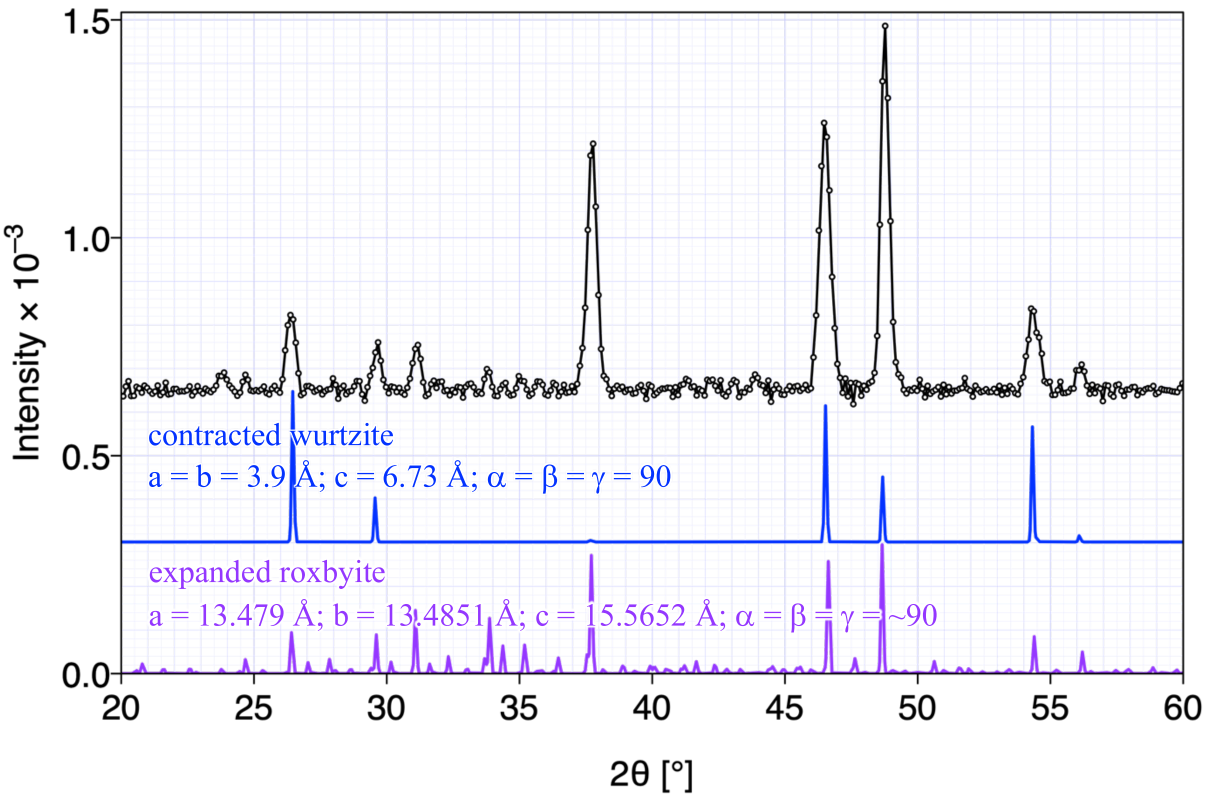

Figure S6. PXRD pattern for Se^{2-} exchanged particles at 100 °C with matched patterns of contracted $Cu_{2-x}Se$ wurtzite and expanded roxbyite $Cu_{2-x}S$ structures.

Table S2. Analysis of the anion exchange efficiency based on SEM-EDS and XRD assuming Vegard's law. To estimate the exchange efficiency based on PXRD data, we assumed that Vegard's law[11] would be valid and give a linear fit between the percent of Se. Thus, we fit the roxbyite $Cu_{2-x}S$ to the wurtzite structure (0% Se) and used this and the known 100% Se wurtzite structure, then fit the a/b lattice parameter to give the equation a/b parameter = 1.4×10^{-3} x %Se + 3.90. The anion exchange efficiency from the SEM-EDS was calculated by converting the Se/S ratio to a percent of exchange.

	a/b lattice parameter (Å)	c lattice parameter (Å)	Anion exchange efficiency calculated from XRD	Anion exchange efficiency calculated from SEM-EDS
Wurtzite $Cu_{2-x}S$	4.04 (from ref. 5)	6.89 (from ref. 5)	100	
260 °C	3.95	6.72	36	30
180 °C	3.94	6.74	29	25
150 °C	3.91	6.73	7	6.5
100 °C	3.90	6.73	0	0
Roxbyite $Cu_{2-x}S$ fit to wurtzite phase	3.90	6.70	0	

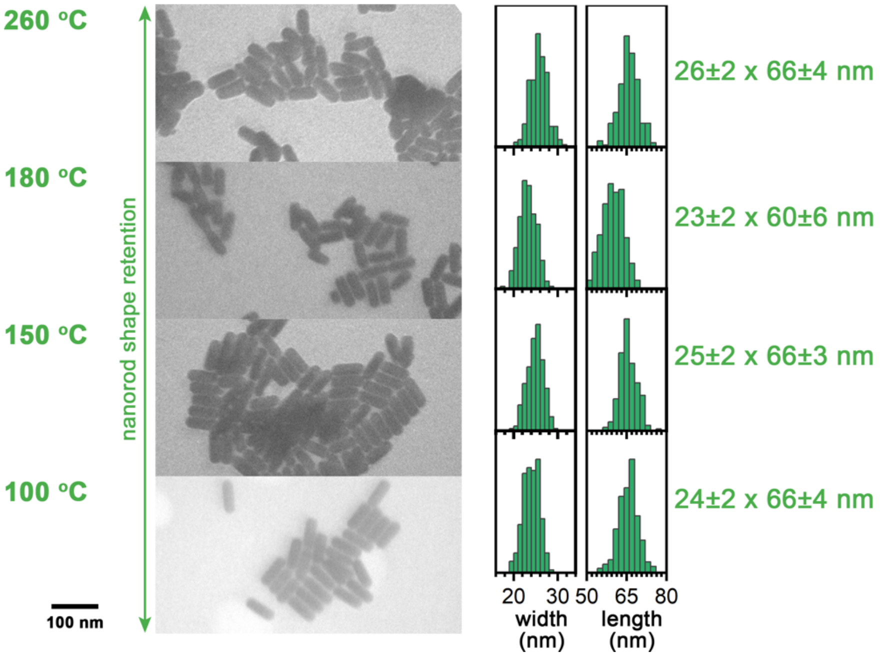

Figure S7. Population analysis of length and width of nanorods after PST with $(C12Se)_2$ demonstrating size and shape retention.

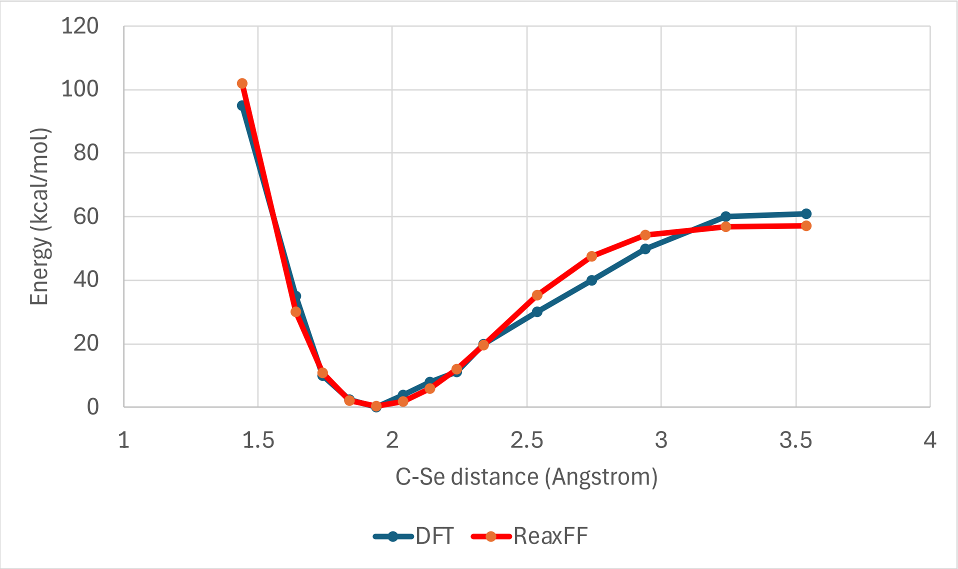

Figure S8. DFT and ReaxFF energies for the C-Se bond dissociation in H_3C-SeH. Note that the 3.5 Angstrom point is from a triplet spin state, while the other data points are derived from the singlet spin state.

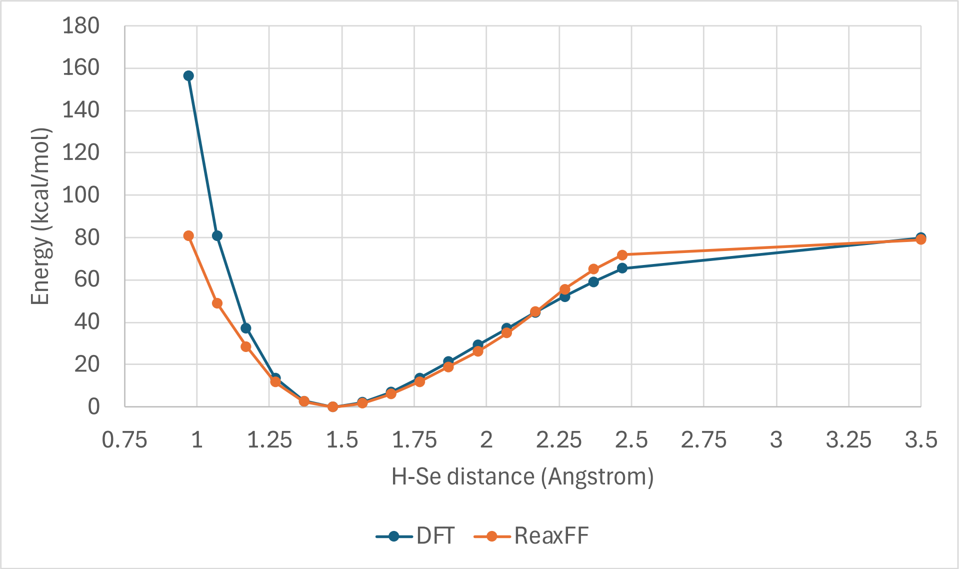

Figure S9. DFT and ReaxFF energies for the H-Se bond dissociation in H_2Se. Note that the 3.5 Angstrom point is from a triplet spin state, while the other data points are derived from the singlet spin state.

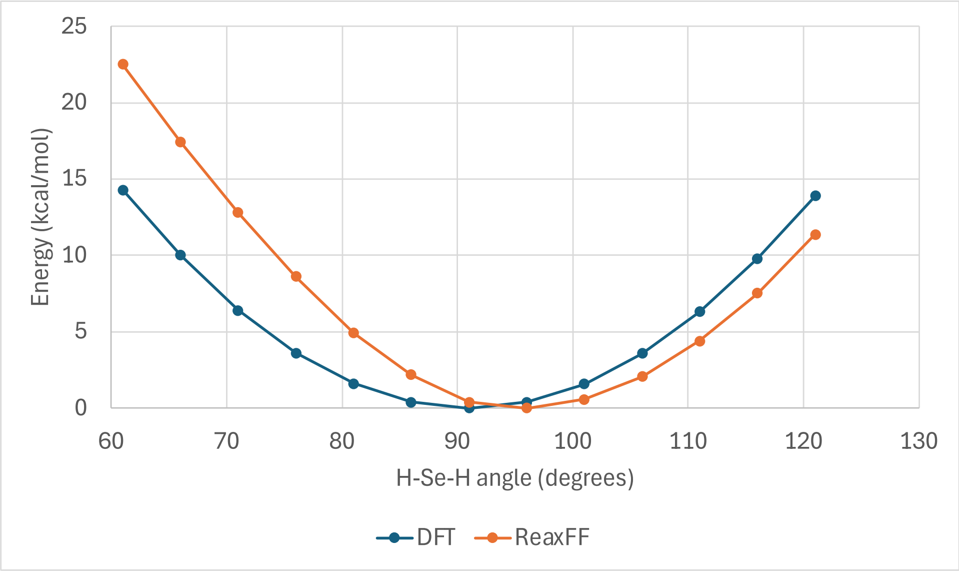

Figure S10. DFT and ReaxFF energies for the H-Se-H angle distortion in H_2Se. All the DFT data points are derived from the single spin state.

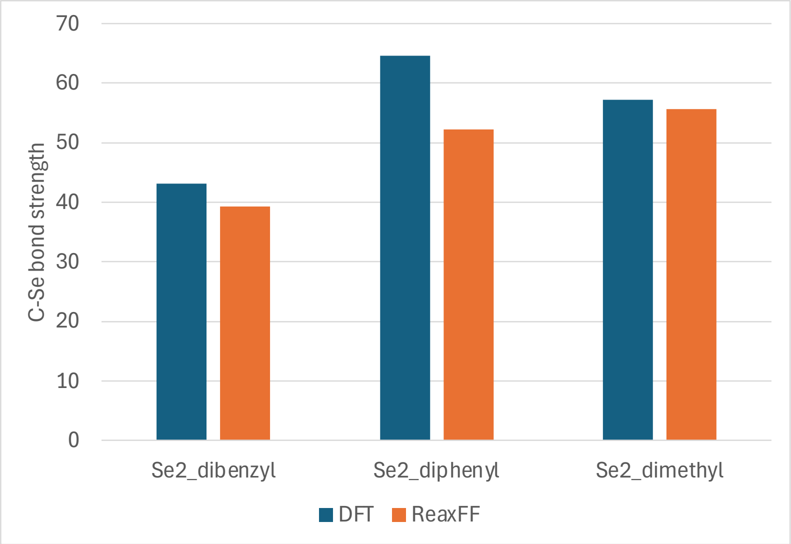

Figure S11. ReaxFF and DFT C-Se bond dissociation energies in diorganyl diselenide molecules.

Table S3. C-Se and Se-Se bond strengths and the difference between then in $(BzSe)_2$, $(CH_3Se)_2$, and $(PhSe)_2$ as calculated by the optimized ReaxFF force field and reported from DFT calculations. [5,6] Note that Guo et al. employed the Boese–Martin Kinetics (BMK) functional with the 6-311G(d,p) basis set in GAMESS. In Tappan et al., geometry optimizations were performed using the 6-31G(d) basis set and the BMK functional. Then, single-point energy calculations were carried out with the 6-311G(d,p) basis set and the BMK functional.

		**C-Se (kcal/mol)**	**Se-Se (kcal/mol)**	**(C-Se) – (Se-Se) (kcal/mol)**
**$(BzSe)_2$**	Tappan et al.	43.09	53.31	-10.22
	ReaxFF	47.25	54.36	-7.11
**$(PhSe)_2$**	Tappan et al.	64.65	42.1	22.55
	Guo et al.	64.44	43.63	20.8
	ReaxFF	65.31	47.05	18.26
**$(CH_3Se)_2$**	Tappan et al.	57.18	54.06	3.12
	Guo et al.	53.76	51.96	1.82
	ReaxFF	56.99	52.12	4.87

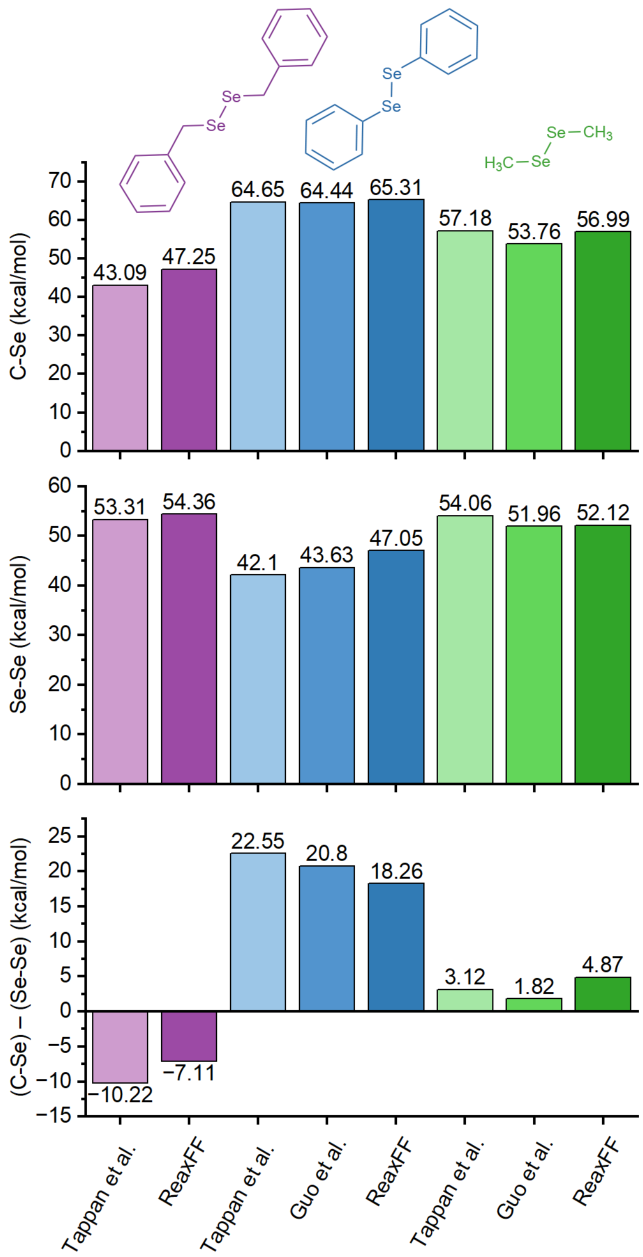

Figure S12. C-C and C-Se bond strengths (in kcal/mol) in $(BzSe)_2$, $(CH_3Se)_2$, and $(PhSe)_2$ as calculated by the optimized ReaxFF force field and reported from DFT calculations.[5,6]

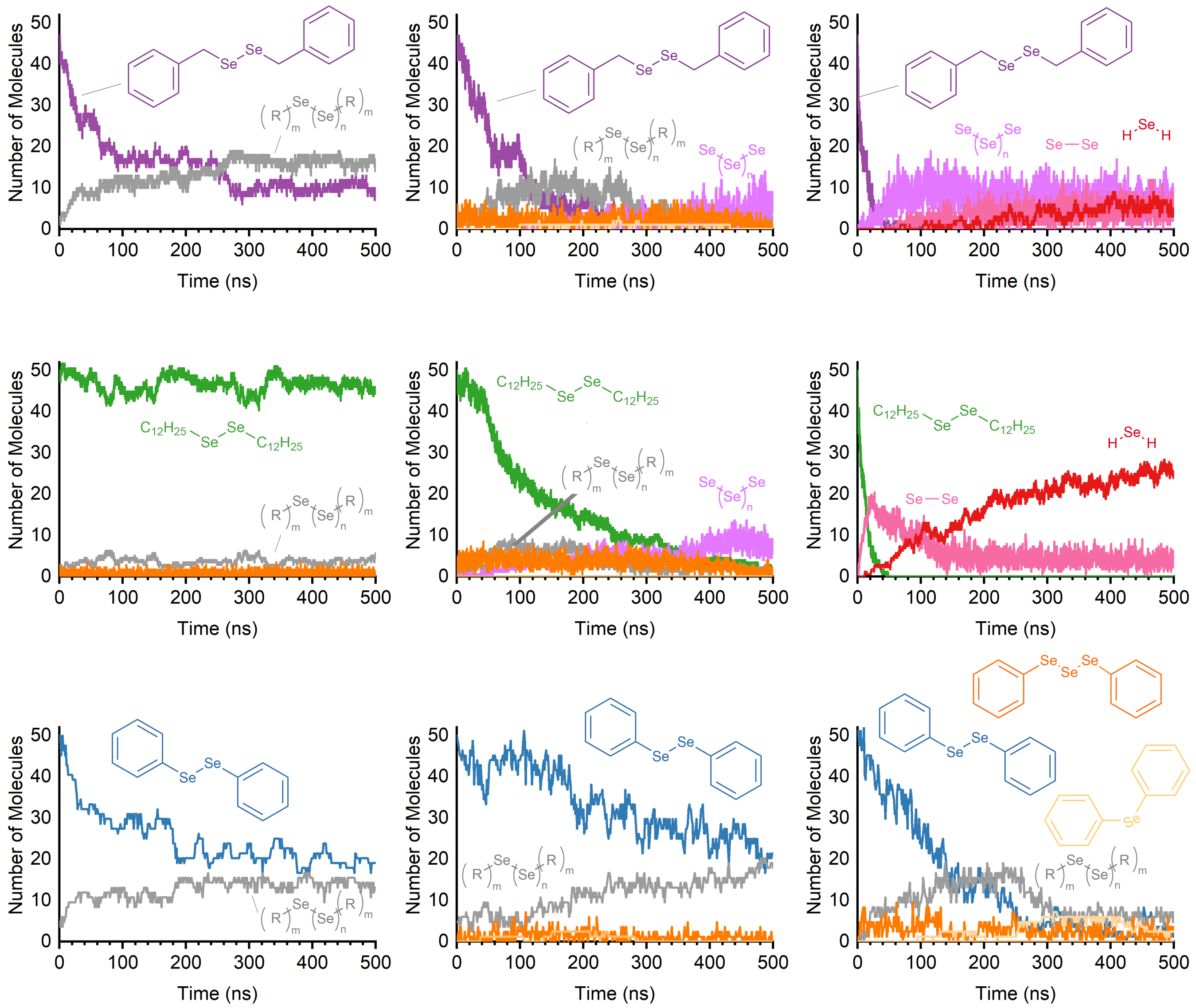

Figure S13. Plot of the Se-containing species formed via MD simulations of thermal decomposition of $(BzSe)_2$, $(C12Se)_2$, and $(PhSe)_2$ at 1000 K, 1500 K, and 2000 K. Oligomer refers to the oligomerization of diorganyl diselenide via Se-Se bonding while maintaining the Se-R bond. Oligoselenides refers to oligomerization of elemental selenium to form polyselenide-like structures.

Table S4. Listing of the species formed via MD simulations of thermal decomposition of $(BzSe)_2$, $(C12Se)_2$, and $(PhSe)_2$ at 1000 K, 1500 K, and 2000 K, including the number of molecules observed throughout the simulation. Only species that made up more than 1% by mass were examined.

	**$(BzSe)_2$**		**$(C12Se)_2$**		**$(PhSe)_2$**	
**1000 K**	Molecule	Maximum #	Molecule	Maximum #	Molecule	Maximum #
	$C_{14}H_{14}Se_2$	47	$C_{24}H_{50}Se_2$	91	$C_{12}H_{10}Se_2$	42
	$C_{28}H_{28}Se_4$	9	$C_{48}H_{100}Se_4$	10	$C_{24}H_{20}Se_4$	8
	C_7H_7	7	$C_{12}H_{25}$	8	$C_{36}H_{30}Se_6$	8
	$C_{42}H_{42}Se_6$	6	$C_{12}H_{25}Se_2$	6	C_6H_5	5
	$C_{35}H_{35}Se_6$	5	$C_{60}H_{125}Se_6$	4	$C_{30}H_{25}Se_5$	4
	$C_{21}H_{21}Se_3$	4	$C_{72}H_{150}Se_6$	3		
	$C_{35}H_{35}Se_5$	3	$C_{36}H_{75}Se_2$	3		
	$C_{26}H_{28}Se_6$	3				
**1500 K**	Molecule	Maximum #	Molecule	Maximum #	Molecule	Maximum #
	$C_{14}H_{14}Se_2$	47	$C_{24}H_{50}Se_2$	91	$C_{12}H_{10}Se_2$	43
	$C_{14}H_{14}$	24	$C_{48}H_{10}Se_4$	10	$C_{18}H_{15}Se_3$	13
	C_7H_7	22	$C_{12}H_{25}$	8	C_6H_5	7
	Se_3	14	$C_{12}H_{25}Se_2$	6	$C_{24}H_{20}Se_4$	6
	$C_{14}H_{14}Se_3$	8	$C_{60}H_{125}Se_6$	4	$C_6H_5Se_2$	6
	$C_{14}H_{21}Se_2$	8	$C_{36}H_{75}Se_2$	3	$C_{30}H_{25}Se_5$	5
	$C_{21}H_{21}$	7	$C_{72}H_{150}Se_6$	3	$C_{12}H_{10}Se_3$	4
	$C_7H_7Se_3$	7			$C_{36}H_{30}Se_6$	3
	$C_7H_7Se_2$	7			C_6H_5Se	2
	$C_{21}H_{21}Se_3$	6				
	$C_{28}H_{28}Se_3$	4				
	$C_{14}H_{14}Se$	3				
**2000 K**	Molecule	Maximum #	Molecule	Maximum #	Molecule	Maximum #
	$C_{14}H_{14}Se_2$	47	$C_{12}H_{25}$	108	$C_{12}H_{10}Se_2$	44
	C_7H_7	39	$C_{24}H_{50}Se_2$	88	C_6H_5	14
	$C_{14}H_{14}$	17	C_2H_4	55	C_6H_6	11
	Se_3	14	C_3H_6	55	$C_{18}H_{15}Se_3$	9
	C_7H_8	10	H_2Se	50	$C_{12}H_{10}Se_3$	7
	H_2Se	9	$C_{12}H_{24}$	34	$C_6H_5Se_2$	7
	HSe_4	8	C_4H_8	33	$C_{12}H_{10}Se$	6
	Se_4	7	Se_2	32	$C_{24}H_{20}Se_4$	5
	HSe_3	7	$C_{12}H_{26}$	22	C_6H_6Se	4
	Se_5	6	H_2	20	$C_{18}H_{15}Se_2$	4
	$C_{21}H_{20}$	5	Se_3	17	$C_{24}H_{20}Se_3$	4
	HSe	5	C_5H_{10}	15	$C_6H_5Se_3$	4
	Se_2	5	$C_{10}H_{21}$	15	C_6H_5Se	4
	Se_6	5	C_8H_{16}	14	$C_{12}H_{10}$	3
	$C_{14}H_{13}$	4	C_9H_{19}	12	$C_{18}H_{15}Se_4$	3

	$C_{21}H_{21}$	4	C_6H_{12}	12	$C_{18}H_{14}Se_2$	2
	$C_{35}H_{34}$	3	C_9H_{18}	11		
	C_6H_6	2	C_7H_{14}	10		
	H_2	2	$C_{12}H_{23}$	9		
	$C_{13}H_{12}$	2	C_6H_{14}	9		
			C_8H_{17}	9		

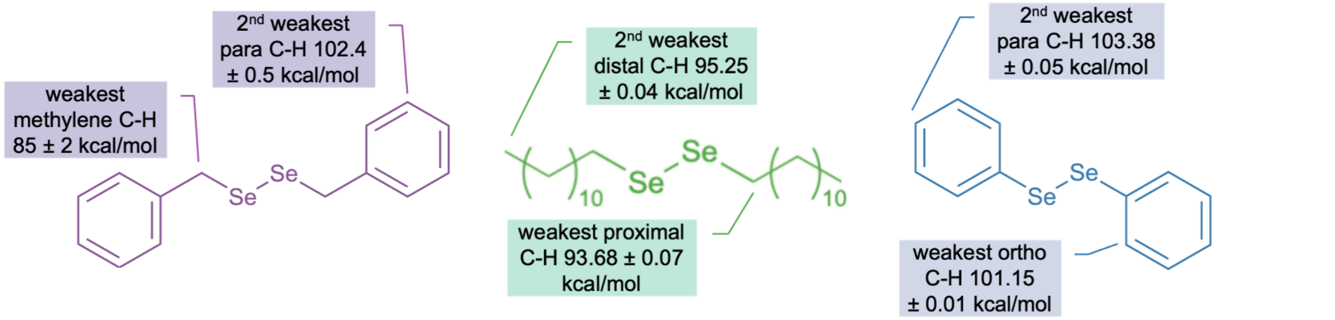

Figure S14. C-H bond strengths in $(BzSe)_2$, $(C12Se)_2$, and $(PhSe)_2$ as calculated by the optimized ReaxFF force field. Only the species with weak C-H bond (<96 kcal/mol) formed H_2Se, with $(C12Se)_2$ producing the greatest amount due to the large amount of H released to protonate Se.

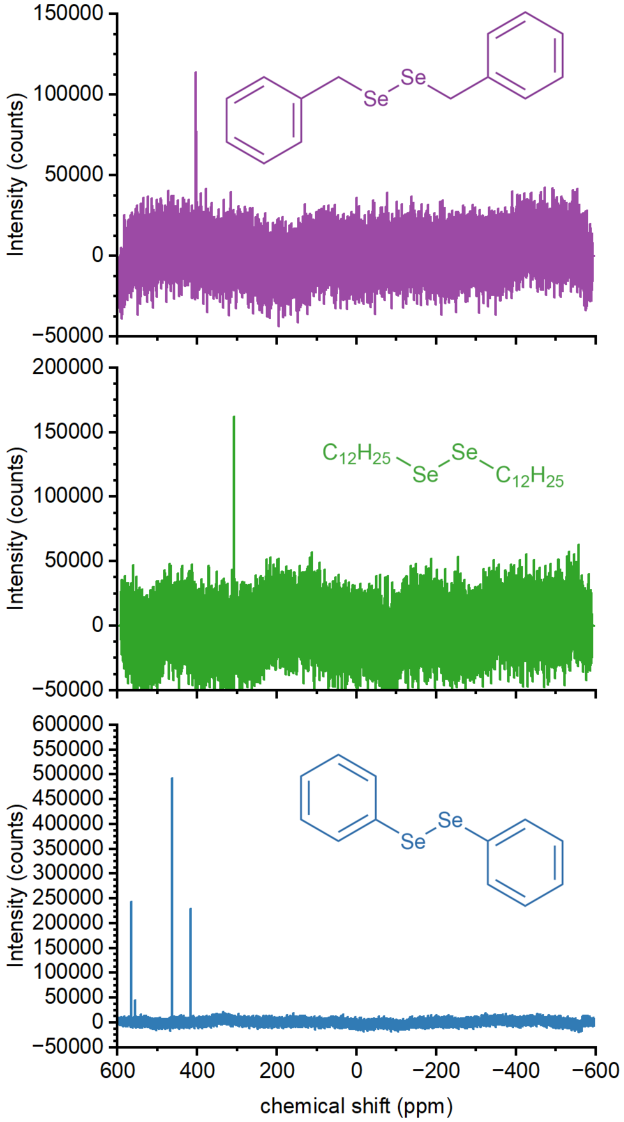

Figure S15. Expanded view of ^{77}Se NMR from thermal decomposition of $(BzSe)_2$, $(C12Se)_2$, and $(PhSe)_2$ at 260 °C showing the intensity values. Note that despite having the same concentration of diselenide initially present, the intensities of the peaks after thermal decomposition for $(BzSe)_2$ and $(C12Se)_2$ are noteably lower than for $(PhSe)_2$. This is due to transformation of $(BzSe)_2$ to solid Se and transformation of $(C12Se)_2$ to H_2Se(g) which is then lost to the headspace and detected by the lead acetate paper.

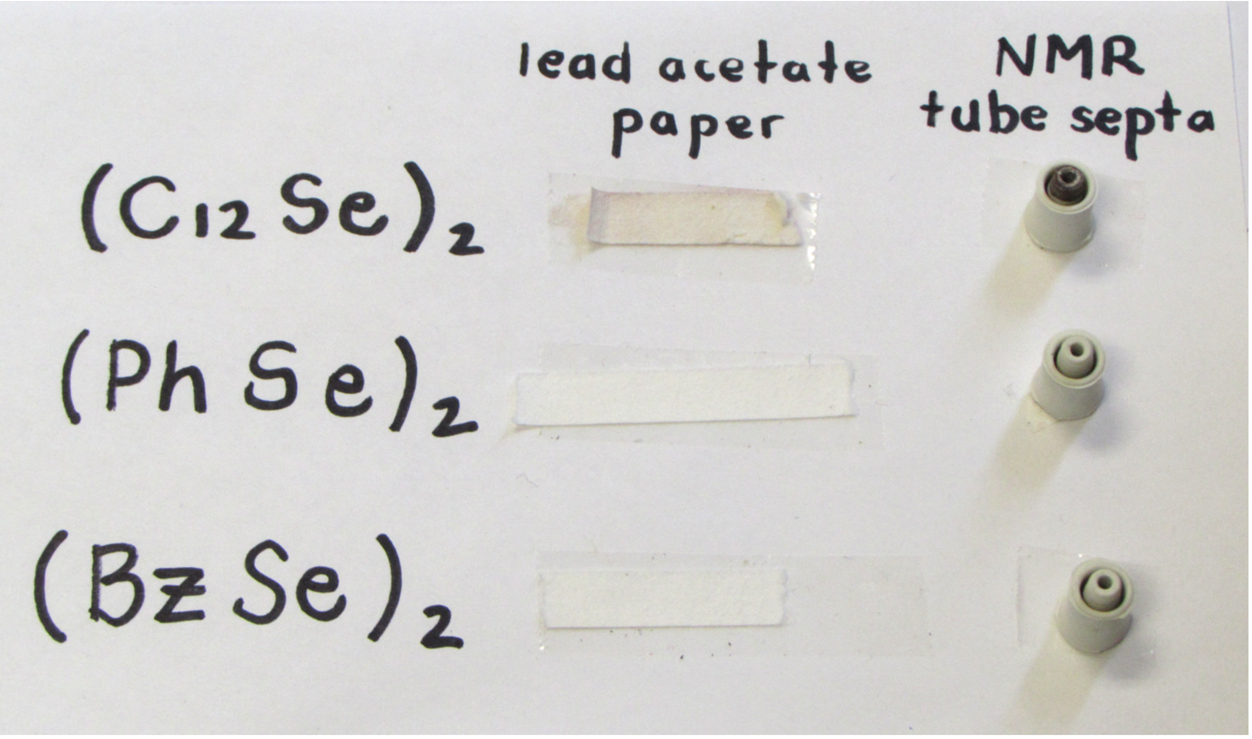

Figure S16. Pictures of the lead acetate tape and rubber septa exposed to the head space during thermal decomposition of $(BzSe)_2$, $(C12Se)_2$, and $(PhSe)_2$ at 260 °C. The discoloration of the tape and septa for $(C12Se)_2$ are indicative of release of H_2Se gas.

Table S5. Listing of the species formed via MD simulations of thermal decomposition of $(C10Se)_2$ and $(C10)_2Se$ at 1000 K, 1500 K, and 2000 K, including the number of molecules observed throughout the simulation. Only species that made up more than 1% by mass were examined.

**$(C10)_2Se$**					
**2000 K**		**1500 K**		**1000 K**	
**Molecule**	Maximum #	Molecule	Maximum #	Molecule	Maximum #
**$C_{20}H_{42}Se$**	50	$C_{20}H_{42}Se$	50	$C_{20}H_{42}Se$	50
**$C_{10}H_{21}$**	27	$C_{10}H_{21}$	25	$C_{10}H_{21}$	5
**$C_{10}H_{20}$**	25	$C_{10}H_{20}$	13	$C_{10}H_{21}Se$	4
**H_2Se**	21	$C_{10}H_{22}Se$	13	$C_{30}H_{63}Se_2$	1
**$C_{10}H_{21}Se$**	19	$C_{10}H_{21}Se$	11	$C_{40}H_{84}Se_2$	1
**$C_{10}H_{22}Se$**	16	$C_{10}H_{20}Se$	4		
**$C_{10}H_{22}$**	15	$C_{20}H_{42}Se_2$	4		
**C_3H_6**	15	$C_{20}H_{41}Se$	4		
**C_4H_8**	12	$C_{10}H_{22}$	3		
**C_2H_4**	11				
**H_2**	11				
**$C_{10}H_{20}Se$**	8				
**$C_{10}H_{19}$**	6				
**C_5H_{10}**	5				
**C_6H_{12}**	5				
**C_9H_{18}**	5				
**CH_2Se**	5				
**C_4H_{10}**	4				
**C_6H_{14}**	4				
**C_7H_{16}**	4				
**$C_{14}H_{30}$**	3				
**C_8H_{16}**	3				

**C10SeSeC10**					
**2000 K**		**1500 K**		**1000 K**	
**$C_{20}H_{42}Se_2$**	50	$C_{20}H_{42}Se_2$	50	$C_{20}H_{42}Se_2$	50
**$C_{10}H_{20}$**	27	$C_{10}H_{21}$	43	$C_{10}H_{21}$	6
**H_2Se**	24	$C_{10}H_{20}$	18	$C_{40}H_{84}Se_4$	5
**C_3H_6**	18	Se_3	14	$C_{30}H_{63}Se_2$	4
**$C_{10}H_{22}$**	15	$C_{10}H_{22}$	11	$C_{60}H_{126}Se_6$	4
**C_4H_8**	14	$C_{10}H_{21}Se_2$	9	$C_{50}H_{105}Se_5$	3
**C_2H_4**	14	$C_{20}H_{42}Se_3$	6	$C_{50}H_{105}Se_6$	3
**H_2**	12	$C_{30}H_{63}Se_4$	6	$C_{30}H_{63}Se_3$	3
**Se_2**	11	$C_{20}H_{42}$	5	$C_{40}H_{84}Se_5$	3
**C_2H_6**	10	$C_{30}H_{63}Se_3$	5	$C_{20}H_{42}Se_3$	2
**Se_3**	10	$C_{20}H_{42}Se_4$	5		
**C_6H_{12}**	8	$C_{10}H_{21}Se_3$	5		
**C_7H_{14}**	7	$C_{20}H_{42}Se$	4		

$C_{10}H_{19}$	6	Se_2	4
C_5H_{10}	6	$C_{30}H_{63}Se_2$	4
C_4H_9	6	C_2H_4	3
C_8H_{16}	5		
C_4H_{10}	5		
CH_4	5		
C_7H_{15}	5		
C_7H_{13}	5		
C_2H_5	5		
$C_{20}H_{42}$	4		
C_6H_{14}	3		

Table S6. Bond dissociation energies for even long-chain dialkyl diselenides calculated with ReaxFF.

	C-Se (kcal/mol)	Se-Se (kcal/mol)	ΔBDE (C-Se)-(Se-Se) (kcal/mol)
$C_{10}H_{21}SeSeC_{10}H_{21}$	45.5	45.2	0.3
$C_{12}H_{25}SeSeC_{12}H_{25}$	43.8	43.3	0.6
$C_{14}H_{29}SeSeC_{14}H_{29}$	45.1	44.9	0.2
$C_{16}H_{33}SeSeC_{16}H_{33}$	45.3	45.0	0.4
$C_{18}H_{37}SeSeC_{18}H_{37}$	45.3	44.9	0.4
Average	45.0±0.7	44.6±0.8	0.3±0.1

References

(1) Rüger, R.; Franchini, M.; Trnka, T.; Yakovlev, A.; van Lenthe, E.; Philipsen, P.; van Vuren, T.; Klumpers, B.; Soini, T. *AMS 2023.103, SCM, Theoretical Chemistry, Vrije Universiteit, Amsterdam, The Netherlands*; SCM, 2023.

(2) Baerends, E. J.; Aguirre, N. F.; Austin, N. D.; Autschbach, J.; Bickelhaupt, F. M.; Bulo, R.; Cappelli, C.; van Duin, A. C. T.; Egidi, F.; Fonseca Guerra, C.; Förster, A.; Franchini, M.; Goumans, T. P. M.; Heine, T.; Hellström, M.; Jacob, C. R.; Jensen, L.; Krykunov, M.; van Lenthe, E.; Michalak, A.; Mitoraj, M. M.; Neugebauer, J.; Nicu, V. P.; Philipsen, P.; Ramanantoanina, H.; Rüger, R.; Schreckenbach, G.; Stener, M.; Swart, M.; Thijssen, J. M.; Trnka, T.; Visscher, L.; Yakovlev, A.; van Gisbergen, S. The Amsterdam Modeling Suite. *J. Chem. Phys.* **2025**, *162* (16). DOI: 10.1063/5.0258496.

(3) Nayir, N.; Wang, Y.; Shabnam, S.; Hickey, D. R.; Miao, L.; Zhang, X.; Bachu, S.; Alem, N.; Redwing, J.; Crespi, V. H.; van Duin, A. C. T. Modeling for Structural Engineering and Synthesis of Two-Dimensional WSe_2 Using a Newly Developed ReaxFF Reactive Force Field. *J. Phys. Chem. C* **2020**, *124* (51), 28285–28297. DOI: 10.1021/acs.jpcc.0c09155.

(4) Fazlioglu-Yalcin, B.; Wang, M.; Nayir, N.; Law, S.; van Duin, A. C. T. Atomic Level Insight into the Nucleation of SnSe Thin Films Using Graphene Mask in Molecular Beam Epitaxy: ReaxFF Molecular Dynamics Simulations. *J. Phys. Chem. C* **2024**, *128* (34), 14294–14304. DOI: 10.1021/acs.jpcc.4c03096.

(5) Tappan, B. A.; Barim, G.; Kwok, J. C.; Brutchey, R. L. Utilizing Diselenide Precursors toward Rationally Controlled Synthesis of Metastable $CuInSe_2$ Nanocrystals. *Chem. Mater.* **2018**, *30* (16), 5704–5713. DOI: 10.1021/acs.chemmater.8b02205.

(6) Guo, Y.; Alvarado, S. R.; Barclay, J. D.; Vela, J. Shape-Programmed Nanofabrication: Understanding the Reactivity of Dichalcogenide Precursors. *ACS Nano* **2013**, *7* (4), 3616–3626. DOI: 10.1021/nn400596e.

(7) Hernández-Pagán, E. A.; Robinson, E. H.; La Croix, A. D.; Macdonald, J. E. Direct Synthesis of Novel $Cu_{2-x}Se$ Wurtzite Phase. *Chem. Mater.* **2019**, *31* (12), 4619–4624. DOI: 10.1021/acs.chemmater.9b02019.

(8) Mumme, W. G.; Gable, R. W.; Petricek, V. The Crystal Structure of Roxbyite, $Cu_{58}S_{32}$. *Can. Mineral* **2012**, *50* (2), 423–430. DOI: 10.3749/canmin.50.2.423.

(9) Wang, J.-J.; Xue, D.-J.; Guo, Y.-G.; Hu, J.-S.; Wan, L.-J. Bandgap Engineering of Monodispersed $Cu_{2-x}S_ySe_{1-y}$ Nanocrystals through Chalcogen Ratio and Crystal Structure. *J. Am. Chem. Soc.* **2011**, *133* (46), 18558–18561. DOI: 10.1021/ja208043g.

(10) Yamamoto, K.; Kashida, S. X-Ray Study of the Average Structures of Cu_2Se and $Cu_{1.8}S$ in the Room Temperature and the High Temperature Phases. *J. Solid State Chem.* **1991**, *93* (1), 202–211. DOI: 10.1016/0022-4596(91)90289-T.

(11) Denton, A. R.; Ashcroft, N. W. Vegard's Law. *Phys. Rev. A* **1991**, *43* (6), 3161–3164. DOI: 10.1103/PhysRevA.43.3161.
